# Parametric Representation of Multiple White Matter Fascicles from Cube and Sphere Diffusion MRI

**DOI:** 10.1371/journal.pone.0048232

**Published:** 2012-11-26

**Authors:** Benoit Scherrer, Simon K. Warfield

**Affiliations:** Computational Radiology Laboratory, Department of Radiology Children's Hospital, Boston, Massachusetts, United States of America; UCSF, United States of America

## Abstract

The characterization of the complex diffusion signal arising from the brain remains an open problem. Many representations focus on characterizing the global shape of the diffusion profile at each voxel and are limited to the assessment of connectivity. In contrast, Multiple Fascicle Models (MFM) seek to represent the contribution from each white matter fascicle and may be useful in the investigation of both white matter connectivity and diffusion properties of each individual fascicle. However, the most appropriate representation of multiple fascicles remains unclear. In particular, a multiple tensor representation of multiple fascicles has frequently been reported to be numerically challenging and unstable. We provide here the first analytical demonstration that when using a diffusion MRI acquisition with only one non-zero b-value, such as in conventional single-shell HARDI acquisition, a co-linearity in model parameters makes the precise model estimation impossible. Motivated by this theoretical result, we propose the novel CUSP (CUbe and SPhere) optimal acquisition scheme to achieve multiple non-zero b-values. It combines the gradients of a single-shell HARDI with gradients in its enclosing cube, in which varying b-values can be acquired by modulation of the gradient strength, without modifying the minimum echo time. Compared to a multi-shell HARDI acquisition, our scheme has significantly increased signal-to-noise ratio. We propose a novel estimation algorithm that enables efficient, robust and accurate estimation of the parameters of a multi-tensor model. In conjunction with a CUSP acquisition, it enables *full* estimation of the multi-tensor model. We present an evaluation of CUSP-MFM on both synthetic phantoms and invivo data. We report qualitative and quantitative experimental evaluations which demonstrate the ability of CUSP-MFM to characterize multiple fascicles from short duration acquisitions. CUSP-MFM enables rapid and effective investigation of multiple white matter fascicles, in both normal development and in disease and injury, in research and clinical practice.

## Introduction

**Figure 1:**
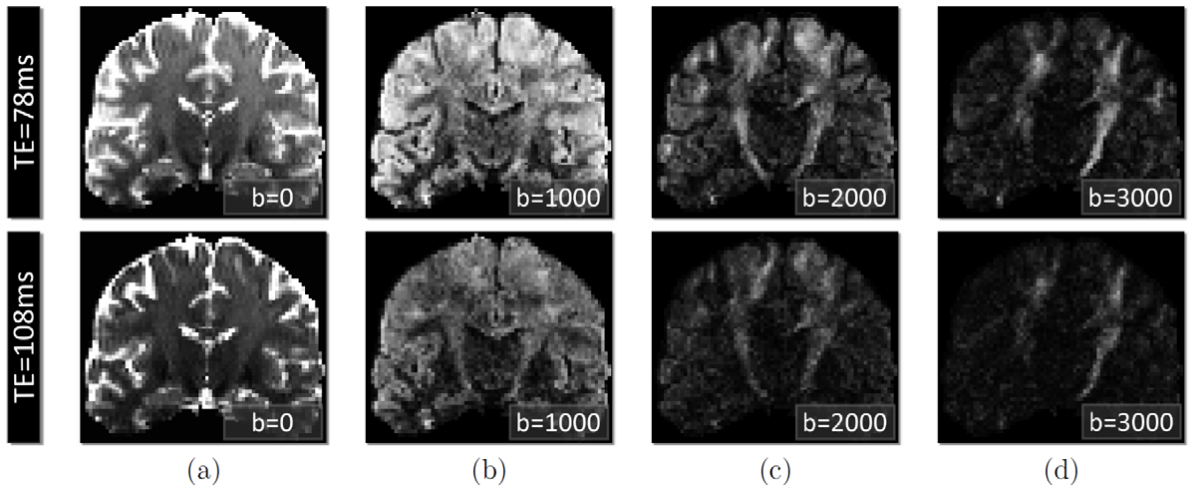
Illustration of the signal decrease when the echo time and the b-value increase in DWI. Diffusion-weighted acquisition with 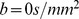 (a), 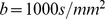 (b), 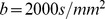 (c) and 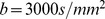 (d). Comparison for 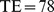
ms (first line) obtained when using our CUSP sequence with 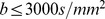, and 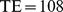
ms (second line) obtained when using multi-shell HARDI sequence with 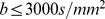. It shows how the signal amplitude decreases (and so does the signal-to-noise ratio) when the b-value *and the TE* increase (first line versus second line). Acquisitions with a short TE should be favored, particularly when imaging at high b-value.

Measuring water diffusion with magnetic resonance diffusion weighted imaging (MR-DWI) has enabled non-invasive investigation and characterization of the white matter architecture and microstructure in the brain. The diffusion in a white matter fascicle has been observed to be highly anisotropic, with primary orientation corresponding to the orientation of the fascicle [1], [2]. The underlying microstructure that gives rise to this anisotropy has been reviewed recently by [3]–[5]. Diffusion tensor imaging (DTI) [6] was proposed to describe the three-dimensional nature of anisotropic diffusion. Assuming *homogeneous Gaussian diffusion* within each voxel, DTI describes the magnitude and orientation of water molecule diffusion with a second-order tensor estimated from diffusion measurements in several directions. More precisely, DTI relates the measured diffusion-weighted signal 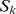 along a gradient direction 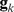 to the non-attenuated signal 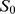 via the Stejskal-Tanner equation [7]:
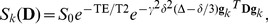
(1)where TE is the echo time, T2 the spin-spin (or transverse) relaxation time of the tissue, 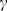 the gyromagnetic ratio, 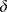 and 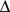 the diffusion sensitizing pulse gradients duration and time separation, and 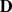 is the 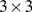 diffusion tensor. The applied b-value defined by 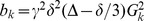, which depends on the gradient strength 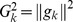, has been introduced [8] to simplify the notations in Eq. 1 and describes the diffusion sensitization strength. The nominal b-value 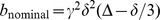 describes the b-value for the unit-norm gradients. The term 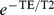 is generally considered constant across all gradients and omitted. However, and importantly, it highlights how the signal amplitude 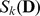 decreases exponentially for increasing TE. A larger TE considerably alters the signal-to-noise ratio for *all* the measurements (see Fig. 1), regardless of the applied b-value. This is essential because minimum achievable TE and nominal b-value are linked. They follow a complex relationship [9], [10] via the timing parameters 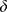 and 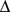, which can be approximated by 
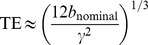
 [11], [12]. Consequently, increasing the nominal b-value increases the minimum achievable TE, which in turn leads to an exponentially decreased signal amplitude closer to the noise floor (see Fig. 1). Considering that the noise amplitude is constant, this signal dropout leads to a lower SNR for each DW image, regardless of their b-value [13]. This leads to a fundamental trade-off in diffusion imaging: while higher b-values are known to increase the contrast *between* the DW gradient directions [14], and therefore to increase the reliability of estimation of orientation of each fascicle, the higher nominal b-value also leads to a longer TE and to a lower SNR *for each DW image*, decreasing the estimation certainty and quality. An optimal diffusion-weighted acquisition must achieve a trade-off between acquiring adequate b-values while minimizing the TE to maximize the SNR.

DTI and its underlying mono-exponential signal attenuation assumption are generally considered to satisfactorily represent *single* fascicles when imaging with b-values lower than 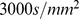 [15]–[17], which is frequently the case in clinical settings. Non-monoexponential behavior of the signal at a voxel in this b-value range can arise from CSF partial voluming [18], mixtures of fascicles present in the voxel [19] and other sources [4]. The diffusion tensor enables representation of the orientation of a single fascicle as well as the characterization of the diffusion process. Tensor parameters such as the fractional anisotropy (FA), the mean diffusivity (MD), the axial diffusivity (AD) and the radial diffusivity (RD) can be computed and have been shown to provide valuable information that reflects changes in the white matter due to development, disease and degeneration. DTI requires relatively short acquisition times and has been successfully employed in clinical studies.

DTI is however well known to be a poor parametric model for representing the diffusion signal arising at voxels that encompass multiple fascicles with heterogeneous orientation such as fascicle crossing, kissing or fanning. A wide number of approaches have been investigated to overcome this fundamental limitation. They involve both novel *diffusion signal sampling schemes* and novel *ways to analyze the diffusion signal* as detailed below.

### Image acquisition strategies

Mainly two q-space sampling strategies have been used for complex fiber structure assessment: Cartesian sampling and spherical sampling. Cartesian sampling is used by diffusion spectrum imaging (DSI) [20], [21]. However, it requires an extremely high number 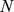 of measurements, typically 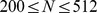, preventing the technique from being used in routine clinical practice. Spherical sampling as employed in high angular resolution imaging (HARDI) techniques reduces the imaging time and requires moderate imaging gradients intensity. A large number of HARDI-based techniques have been proposed (see next section). Note that in this work, to avoid any confusion between the image acquisition strategy and the signal modeling strategy, we denote by HARDI the *acquisition scheme* only. Single-shell HARDI acquisitions with a single non-zero 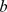-value have been considered to image a sphere of constant radius in q-space. Multiple-shell HARDI acquisitions have also been proposed. They combine in a single acquisition the sampling of multiple shells of different radius in q-space. It enables acquisition of multiple non-zero 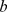-values. Multiple-shell HARDI, however, leads to a large TE that depends upon the highest b-value. This leads in turn to a significantly lower signal-to-noise ratio (SNR) for all the measurements (see Eq. 1 and [22]) and to a longer imaging time. In addition, imaging a higher b-value is generally achieved by using longer diffusion gradient pulse duration, which in turn leads to larger eddy current distortion [23], [24].

Other sampling techniques have been proposed for reasons other than assessing complex fiber structures. Sampling using the tetrahedral 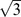-norm gradients has been employed [9] to measure the apparent diffusion coefficient (ADC) from four diffusion measurements. Because 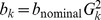, it enables imaging at higher b-value than the nominal b-value *without* modifying the timing parameters 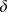 and 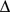, but by using *gradients with norm greater than one*. It provides the optimal minimum achievable TE for the corresponding applied b-value, leading to a better SNR and potentially to lower eddy current distortion because the diffusion gradient pulses can be shortened. Using the same concept [25], employed the six hexahedral 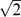-norm gradients to estimate a diffusion tensor from seven measurements. Furthermore, in CURVE-Ball (CUbe Rays to Vertices and Edges) [26], a spherical sampling and the hexahedral [25] and tetrahedral [9] gradients were combined to perform the estimation of a single-tensor model at three different diffusion scales 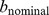, 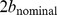 and 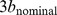.

### Models for characterization of the diffusion signal

A large number of approaches have been investigated to analyze the diffusion signal and represent multiple white-matter fascicles with complex geometry. Both parametric (model-based) and non-parametric (model-free) approaches have been proposed. They generally focus on estimating either (1) the diffusion displacement probability density function (diffusion PDF), (2) the diffusion orientation distribution function (dODF) which is the angular profile of the diffusion PDF or (3) the fiber orientation distribution function (fODF), also known as the fiber orientation density (FOD) and which is of central interest for tractography.

Model-free approaches include diffusion spectrum imaging (DSI) [20], [21]. In this technique, the diffusion PDF is directly estimated from the inverse Fourier transform of the measured signal, requiring a very high number of measurements to satisfy the Nyquist condition. Q-ball imaging (QBI) [27] estimates an approximate non-parametric angular profile of the diffusion PDF without actually computing the diffusion PDF, by using the Funk-Radon transform. Fast and robust analytical QBI estimation procedures have been proposed [28]–[31]. However, QBI results in the estimation of an approximated dODF related to the true dODF by modulation with a zero-order Bessel function. This leads to a spectral broadening of the diffusion peaks of individual fascicles at moderate b-values accessible on a clinical scanner, perturbing the FOD reconstruction necessary for carrying out tractography. Mixing of individual tracts in a voxel leads to local maxima that do not coincide with the true fascicle orientation [32], leading to a relatively low fidelity representation. To avoid the usual Q-Ball approximation, Canales-Rodríguez *et al.* [33] have derived in Exact Q-Ball Imaging (EQBI) a direct relationship between the dODF and the diffusion data. Its enables the estimation of the exact dODF under the assumption of a Gaussian profile.

Q-space approaches such as DSI, QBI, or EQBI are however limited by three major error sources. First they are based on the *narrow pulse approximation* assumption, considering that molecules do not diffuse during the application of the diffusion sensitizing gradients. The gradient pulses are then modeled by a Dirac shape which is not practically feasible, especially on clinical systems. In practice, in clinical settings, the diffusion-encoding gradient duration 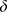 is typically of the same order of magnitude as the time offset 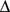 between encoding gradients [34] (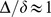) to minimize 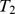 decay and to obtain better SNR, which is a very poor approximation of a Dirac shape. Second, since the imaging time has to be finite, only a finite region in q-space is imaged. This has been shown to lead to a blurred propagator with decreased contrast and angular resolution [35]. Third, they are limited by the need to truncate the Fourier representation which is required to numerically compute the infinite series involved in the Fourier transformation, leading to quantization artifacts [33].

In contrast, parametric models describe a predetermined model of diffusion rather than an arbitrary one. They potentially require a smaller number of images to be acquired, leading to a reduced acquisition time. A large number of model-based approaches have been investigated. Among them, generalized diffusion tensor imaging (GDTI) [36], [37] models the white-matter fascicles with higher-order tensors ; spherical deconvolution (SD) [38]–[40] directly estimates the FOD instead of the dODF and has a better angular resolution; diffusion orientation transform (DOT) [16] employs a model-based q-space modeling based on the assumption of a monoexponential decay of the signal attenuation.

A major drawback to DSI, QBI, DOT, SD and GDTI is that they focus on describing the *general shape* of the diffusion profile in each voxel. They do not represent each fascicle independently and therefore do not characterize the proportion of each fascicle passing through a voxel. Importantly, they do not enable characterization of each fascicle. Diffusion parameters such as the generalized fractional anisotropy (GFA) can be computed but represent a DW signal dispersion property rather than an individual fascicle property. For example, a synthetic fascicle consisting of an identical tensor at every voxel crossed by another synthetic fascicle has a GFA that varies in the crossing region [41], which is not clinically relevant. It is not possible to distinguish whether a change in diffusion parameters along a fascicle is associated with a change in the intrinsic fascicle property or because of the presence of crossing fascicles. These approaches provide information about the distribution of fascicle *orientations* in the voxel but are limited to connectivity assessment.

In contrast, multi-fascicle models (MFM) consider at each voxel a mixture of independent fascicles with heterogeneous orientation. Making the assumption of a slow exchange between the fascicles' compartments, the diffusion signal in each voxel is modeled as a mixture of the diffusion signal arising from each individual fascicle. Integration of an isotropic component has also been investigated [17], [42]–[45] to model the diffusion of unrestricted water. This enables characterization of pathologies such as edema, stroke or inflammation. This also enables characterization of the CSF contamination [46] due to partial volume effect, known to perturb the accurate estimation of the anisotropic diffusion compartments [18], [47]. Ultimately, the diffusion-weighted signal 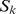 along a gradient direction 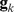 for MFM with an isotropic compartment and 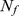 fascicles can be described by the following general mixture:
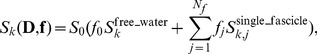
(2)where 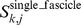 is the diffusion signal arising from a single fascicle, 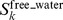 is the diffusion signal arising from the unrestricted water diffusion, and 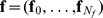 describes the *fractions of occupancy* of each compartment (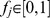) and sum to one.

The diffusivity of free water is generally considered to be well modeled by an isotropic Gaussian distribution [17], [42]–[45], leading to 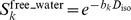 with 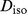 is the diffusivity of free water.

In a particular case of multi-fascicle model, the ball-and-stick model [42], [43], each individual fascicle has been represented by a stick in the expression of 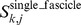. With this simplification, an essential advantage of multi-fascicle models is lost: the ball-and-stick model provides information only about the fascicles orientation. It does not enable the assessment of fascicle properties such as the fascicle anisotropy and diffusivity, limiting the use of the ball-and-stick model to connectivity studies.

In contrast, since an individual fascicle is generally considered to be well represented by a single tensor in DTI, a natural candidate has been to represent each fascicle by a tensor. Considering 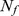 tensors 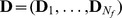 representing the 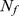 fascicles, this amounts to setting 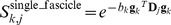, leading to the so-called multi-tensor model [42], [45], [48]–[52]. The multi-tensor model has the fundamental advantage over other common representations of modeling each fascicle independently. It enables the assessment of *individual* fascicle characteristics by computing diffusion tensor parameters for each fascicle. This enables characterization of the white-matter appearance, changes and alterations. This also enables comparison of diffusion characteristics between corresponding anatomical fascicles across individuals, which is of great interest for clinical applications. In addition, the *full* multi-tensor model estimation enables characterization of the fraction of occupancy for *each* fascicle, providing information about the mixing proportions and compensating for partial volume effect.

**Figure 2:**
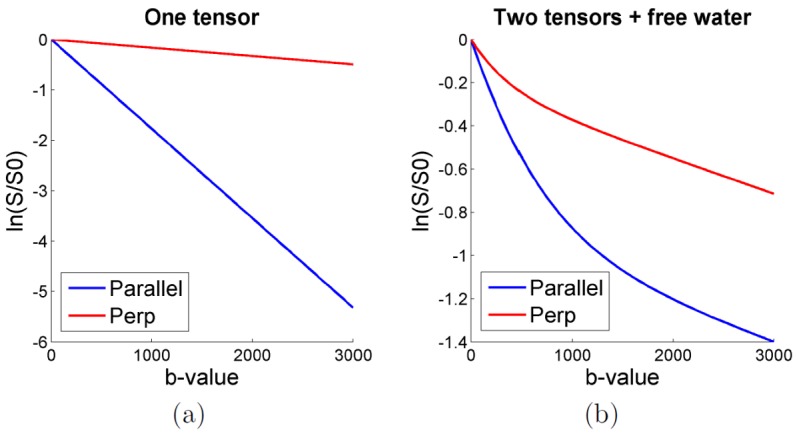
Intra-voxel orientation heterogeniety and partial volume averaging leads to a non-monoexponential decay in a voxel. (a): Illustration of the monoexponential decay arising from a single tensor (FA = 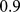, diffusivity = 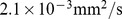) as shown by the linearity of 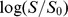 in both the parallel and perpendicular directions with respect to the tensor orientation (noise-free case). (b): Illustration that mixing of an isotropic compartment (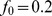, 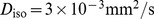) and two crossing fascicles represented by two single tensors (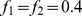, FA = 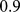, diffusivity = 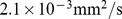, crossing angle = 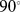) using Equation 2 leads to a non-monoexponential decay *in the voxel*, even for b-values below 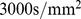. This illustrates that a non-monoexponential decay in a voxel may arise from a sum of mono-exponential behaviors.

Multiple works have pointed out that a non-monoexponential decay may be observed *in voxels* when imaging with high b-values [3]–[5], [15], [6], [53]–[59], providing evidence that the *single tensor model* and its underlying Gaussian assumption is not appropriate to accurately represent the diffusion signal *in the voxel*. The biophysical mechanisms responsible for the non-monoexponential behavior are, however, numerous and not completely understood. First, it is commonly recognized that compartmentalization of the voxel in different subregions with heterogeneous properties can lead to a non-monoexponential decay [3], [4], [56] under certain acquisition conditions. Particularly, as illustrated by Fig. 2, mixing of an isotropic unrestricted water compartment with multiple anisotropic compartments (Equation 2), each of them being modeled with a purely monoexponential decay, leads to a non-monoexponential decay due to partial volume averaging, even at moderate b-values. At a smaller diffusion scale, the presense of intra- and extracellular compartments does lead to a non-monoexponential decay for very high b-values, even for a single fascicle. Nevertheless, the presence of this phenomenon at clinically relevant b-values, with long diffusion sensitization pulse duration 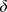, long echo times and low signal-to-noise ratio remains unclear.

Importantly, compartmentalization is not a prerequisite for the presence of a non-monoexponential decay. Schwarcz *et al.* [57] have reported the presence of a biexponential decay in the cold-injured brain parenchyma *after massive membrane disintegration*, and in centrifuged erythrocyte samples. Sehy *et al.* [54] have observed non-monoexponential behavior within the intracellular space of a *single* cell, the frog oocyte. Other biophysical mechanisms, such as the proximity of cell membranes which locally restrict motion, and intra- and inter-cellular heterogeneities, are likely to contribute to the MR signal decay behavior. Imaging strategies that uniquely characterize each of these properties remain under development [3]–[5], [56].

Multiple approaches have been investigated to account for the non-Gaussianity of the diffusion signal in a voxel [5], including fitting a multi-exponential model [4], [16], [53], [58] and a “stretched-exponential model” [55]. Jensen *et al.* [59] have investigated the estimation of a Kurtosis term, which is a dimensionless measure of the deviation of the water diffusion profile from a Gaussian distribution. Assaf *et al.* [15] proposed a ‘composite hindered and restricted model of diffusion’ (CHARMED), in which the diffusion signal was characterized by components arising from hindered (extra-axonal water) and restricted (intra-axonal) water diffusion, featuring a perpendicular diffusion component that is non-monoexponential. CHARMED requires long acquisition times and very high b-values (up to 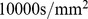), limiting its use in routine clinical practice.

To the best of our knowledge, all approaches accounting for the non-monoexponential signal decay have considered the case of a single fascicle in each voxel. For example, Cheung *et al.* [60] have measured significant deviation from the Gaussian distribution with estimation of a single tensor and a single Kurtosis term with b-values as low as 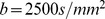. However, as illustrated by Fig. 2, the intra-voxel orientation heterogeneity and the partial volume effect may be the predominant sources of the observed non-monoexponential decay at such diffusion scale. More precisely, while the presence of a non-monoexponential decay *for an individual fascicle* is commonly accepted when using very high b-values and short gradient pulse duration 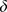, its presence in data acquired with a clinical scanner with limited b-value range and large 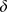 remains unclear. Particularly [15]–[17], suggest that the non-monoexponential behavior is negligible when considering b-values lower than 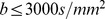, and that when the acquisition time or the available gradient strength is limited, a monoexponential *per-fascicle* model can be safely employed [16].

Therefore, in this work, we focused on a representation of each individual fascicle by a single tensor model. The *full multi-tensor model* estimation enables the assessment of individual fascicles characteristics in addition to the brain connectivity. Diffusion parameters (FA, MD, AD, RD) can be computed for *each* fascicle independently which is of central interest for fascicle integrity assessment. The number of parameters involved is relatively small, requiring a limited number of acquisitions for their estimation. However, full multi-tensor approaches have frequently been reported to be numerically challenging and unstable, experiencing difficulties for their estimation in practice. We show that this is due to inappropriate imaging acquisition settings leading to an under-determined system of equations, and we propose a complete solution.

### Contributions

The contributions of this work are three-fold. First we provide the *theoretical* demonstration that multi-tensor models cannot be fully estimated with a single-shell HARDI acquisition because the tensor size and the fraction of occupancy are collinear, leading to a system of equations with an infinite number of solutions. With a single non-zero b-value, only the tensor orientation can be correctly estimated, but not the tensor size nor the fractions of occupancy. Multiple non-zero b-values are required to ensure a unique solution and to entirely estimate the full multi-tensor model, enabling simultaneous estimation of the tensor orientation, the tensor size and the fractions of occupancy.

Second, we propose a novel multi-tensor optimization technique based on the maximum *a posteriori* (MAP) principle. This allows us to combine the model estimation and the model regularization to reduce the effect of noise. Our prior is based on a finite difference scheme in which only tensors which are part of the same fascicle are regularized together. It is formulated in the log-Euclidean framework, which prevents leaving the set of symmetric positive definite matrices during the optimization and ensures non-degenerate solutions. Our formulation enables efficient optimization of the parameters and enables the introduction of suitable constraints on the estimated tensors.

Third, we propose to employ a novel acquisition scheme that enables estimation of a *full* multi-tensor model with optimal TE and consequently optimal SNR. Our CUbe and SPhere (CUSP) acquisition technique combines a single shell HARDI with images in the enclosing cube of constant echo time. We show that the enclosing cube of the shell is a *cube of constant TE*, in which gradients with varying b-values can be imaged without increasing the TE, by using gradients with norm greater than one. This satisfies the need for multiple non-zero b-values, enabling the estimation of the complete multi-tensor model. It incorporates high b-values which allows for better characterization of multi-compartment models [43], [61]. We propose three ways to construct a CUSP acquisition based on a projected or a truncated multi-shell HARDI. The strength of our imaging technique is to achieve multiple b-values higher than the nominal b-value *while* achieving the same low TE as a single-shell HARDI. Compared to a multiple-shell HARDI, CUSP leads to a significantly higher signal-to-noise ratio, shorter imaging time and to potentially lower eddy current distortion.

The paper is organized as follows. We provide in Section 0.1 the theoretical demonstration that multi-tensor models require multiple non-zero b-values to be fully estimated. We describe our novel algorithm for estimating the parameters of the multi-fascicle model (MFM) in Section 2. We detail our Cube and Sphere (CUSP) imaging technique in Section 0.3. The CUSP-MFM evaluation includes several qualitative and quantitative experiments with both synthetic and in vivo data: angular resolution performance, comparison with the state-of-the-art ball-and-stick model and bootstrap experiments. We show that CUSP-MFM enables the characterization of multiple white-matter fascicles from short duration acquisitions compatible with routine clinical practice.

## Materials

### 1 Theory: demonstration that multiple 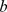-values are required

We demonstrate in this section that the tensors and fractions of occupancy of a multi-tensor model cannot be uniquely determined when using a single shell HARDI acquisition [62]. Consider a model with two fascicles represented by the two diffusion tensors 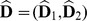 and the fractions 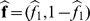, and let consider an acquisition with a unique non-zero 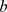-value 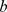. If 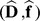 are the underlying true tensors and fractions, then for any 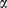, 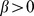 Equation 2 can be written as:
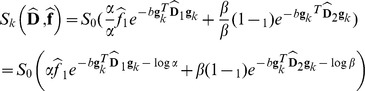
Let 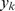 be the measured signal for the direction 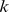 and 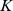 the number of diffusion gradients. 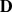 and 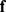 are generally estimated by a least-square approach by considering:
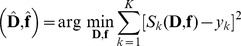
(3)Because 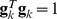, we have 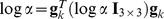 and:
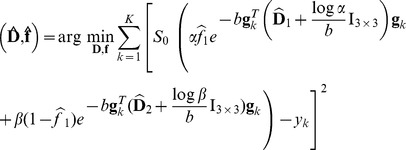
We can show that for 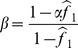 and 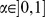 then 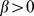 is satisfied and so are the fundamental properties of a mixture model: (1) the fractions sum to one, *i.e.* 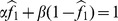 and (2) each fraction is positive and not greater than one, *i.e.* 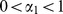 and 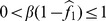.

Consequently, when using a single non-zero 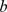-value acquisition, then if 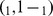 and 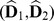 is a solution of Equation 3, then for *any* 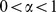, 
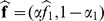
 and 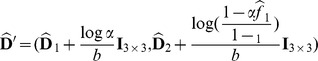 is a solution of Equation 3 as well, because 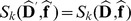 for all 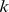. There is an infinite number of solutions. Additionally, non-degenerate tensors are obtained for 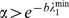, 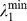 being the minimum eigenvalue of 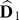. The tensor size indicated by the magnitude of its eigenvalues and the partial volume fractions are collinear and cannot be uniquely determined. Intuitively it indicates that when using a single non-zero b-value, a decrease of the signal modeled by one of the tensors can be compensated for by an increase of the signal modeled by the other tensor, by transforming the tensor diagonals and the fractions.

It is not the case with multiple non-zero b-values 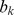 because 
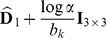
 and 
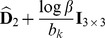
 are function of 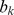. For example, if we consider two b-values 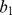 and 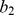, and separate the terms depending on 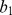 corresponding to the indices 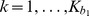 and the terms depending on 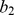 corresponding to the indices 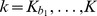, it follows that:
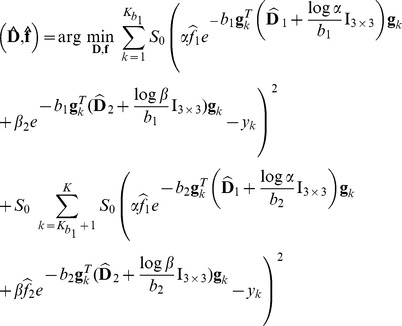
A unique new multi-tensor model 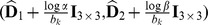 does not satisfy 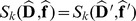 for all 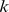 because, in contrast to the single non-zero b-value case, it depends on 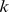. The use of multiple non-zero 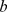-values enables a unique solution to be found and disambiguates the estimation of 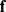 and 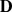
This allows measurements of the fractions of occupancy and of each tensor size and orientation.

### 2 A novel multi-tensor parameter estimation procedure

We consider the image domain 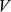 to be a regular 3-dimensional (3D) grid, and consider the *full* multi-tensor model described by :
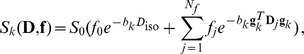
Our aim is to recover the multi-tensor models 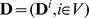 and the fractions 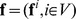 for each voxel of 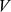. When estimating tensors, particular care must be taken to ensure the positive-definitive property of the 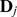 and to avoid degenerate tensors with null or negative eigenvalues. Although such tensors are non-physical, they commonly arise in high anisotropy regions or due to noise corruption [63]. Here we ensure the symmetric positive definite property of each tensor by parameterizing them in the log-Euclidean framework [64], [65], by setting 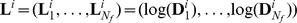. It ensures that tensors with null or negative eigen-values are at an infinite distance. In contrast to Euclidean approaches, it does not require any particular care to preserve tensor attributes during the computation because all operations are performed within the appropriate manifold.

We denote by 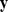 the set of gradient images, with 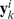 denoting the 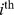 voxel of the gradient image 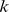. The simultaneous estimation and regularization of 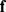 and 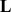 (and consequently 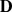) is performed according to a maximum *a posteriori* principle, by maximizing:
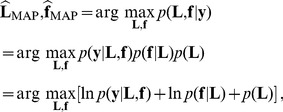
(4)which decomposes into a likelihood term and two prior terms. We assume statistical independence of the noise between the images and between the voxels, so that 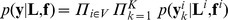. Furthermore, we assume a Gaussian noise with zero-mean and variance 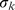, and consider the following likelihood:
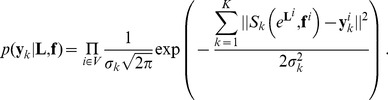
(5)The term 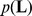 in Eq.4 enables us to incorporate a prior knowledge on the multi-tensor field 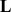. In this work we consider an anisotropic regularization prior that exploits spatial homogeneity but preserves sharp contours. More precisely, we favor smoothness of each 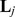 by setting 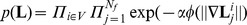 where 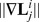 is the norm of the spatial gradient of 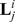, and 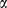 is a parameter controlling the regularization strength. As generally employed, we set 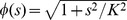 to account for *anisotropic* regularization, K being a normalization factor for the gradient. Following the one-tensor log-Euclidean model of [64] we set 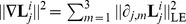 with 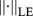 the log-Euclidean metric [65]. The regularization of multiple tensor models is, however, *not* the straight extension of the single-tensor case. Rather, only tensors which are part of the same fascicle should be regularized together. To achieve this, we propose an original approximation of the spatial gradient for the multiple tensor case. The partial derivative in a direction 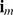 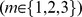 is approximated by considering the two most similar neighbors 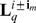 to 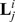 in the following finite difference scheme:
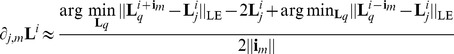
(6)Note that this formulation is compatible when regularizing neighboring voxels containing a different number of tensors. A *softmax* approximation of the 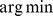 operator can be considered to ensure the differentiability of the regularization term. Indeed, by considering a finite set of measures 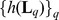, 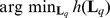 can be approximated by:
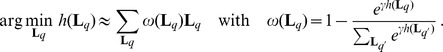
This expression ensures 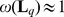 for the smallest 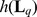 and 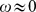 for the others. Choosing a large value for 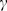 allows faster transitions of 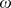 between 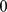 and 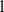.

In this work we did not considered any prior knowledge on the estimated fractions and considered 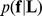 (see Eq.4) to be a uniform density. Ultimately, by considering constant noise characteristics across acquisitions, maximization of the posterior distribution in Eq.4 leads to the following minimization:
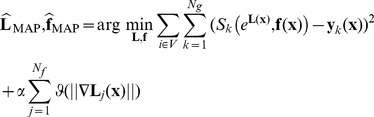
(7)


#### Euler-based parameterization of the tensor orientations

We parameterize each tensor's orientations with the Euler angle. We experimentally found this representation to enable a more efficient optimization of the parameters. In addition, it enables the choice of introducing various constraints to further reduce the number of parameters: symmetry of the eigenvalues (*e.g.* 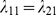), cylindrical shape of each tensor (*e.g.* 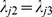), bounds on the magnitude of the eigenvalues, equiplanar configuration for the tensors, and others.

#### Initialization

The model parameters are estimated by performing an iterative minimization which requires a starting point. As in [50] we initialize the multi-tensor fitting procedure by considering the one-tensor solution obtained by a robust least-squares estimate. We denote by 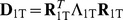 the one-tensor solution with eigen values 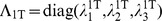. To enable a faster convergence, the first two tensors 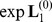 and 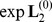 are initialized according to the rotation of 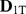 of angle 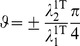. The rotation is applied in the plane formed by the two largest eigen values 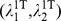 and composed with a shrink of 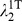. In consequence, when 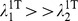, which is likely to indicate an individual fascicle in that voxel, the initial 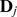
's are two tensors with almost parallel principal diffusivities. In contrast, when 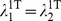, the initial 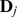
's describe two tensors whose principal diffusivities are perpendicular. When estimating more than two tensors, the orientation of 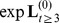 is initialized with a random rotation of 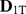.

#### Numerical optimization

The solution of Equation 7 is obtained using the BOBYQA algorithm [66], a recent derivative-free bound-constrained optimization technique. BOBYQA is an iterative algorithm. At each iteration, it computes from a set of points a quadratic approximation for the objective function. The point giving the largest value for the objective function is replaced by the minimum of the quadratic approximation computed in a trust region. At each iteration the trust region is updated. BOBYQA converges faster than the Newton method and enables the introduction of constraints. The introduction of constraints on the 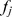
's enables the estimation of properly bounded fractions of occupancy (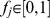), while constraints on the Euler angles ensures the uniqueness of the Euler parameterization. We found it to be less sensitive to local minima than a conjugate gradient descent scheme. The numerical optimization is achieved by considering a diffusion model with gradually increasing complexity, starting from a simple stick model and finishing by the estimation of the *full* multi-fascicle model including the fractions of occupancy, the tensor orientations, the tensor eigen-values and the unrestricted water compartment. This makes the minimization less sensitive to the initialization, providing a robust full MFM estimate at each voxel.

### 3 The CUSP gradient encoding scheme

We have demonstrated in Section 0.1 that multiple non-zero b-values are required to fully estimate multi-tensor models. In this section we provide an optimal gradient encoding scheme which satisfies this requirement.

In diffusion weighted imaging, a key parameter in controlling the signal-to-noise ratio (SNR) is the echo time (TE). An increase in TE leads to a signal dropout due to T2 relaxation and therefore to a decrease in SNR (see [22], Eq. 1 and Fig. 1). Keeping the TE as low as possible is essential to acquire high quality measurements. However, the TE cannot be set to an arbitrary value, but is constrained by the choice of the nominal b-value 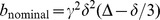 of the acquisition. The minimum achievable TE follows a complex relationship with 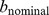 [9], [10] which can be approximated [12] by:
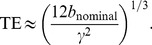
(8)An ideal acquisition scheme for the estimation of a full multi-tensor model should (1) achieve multiple non-zero b-values and (2) achieve the optimal trade-off between imaging high b-values and minimizing the TE to maximize the SNR.

Single-shell HARDI as used in [19], [45], [48]–[50] to estimate a multi-tensor model employs gradients of constant strength 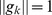 for each direction and provides a single-radius spherical sampling in q-space. Because the applied b-value is 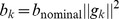, a single-shell HARDI acquires only a single non-zero b-value equal to 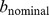, which is not suited for the full estimation of multiple tensors. Separate single-shell HARDI scans with different nominal b-values can be employed to image multiple non-zero b-values. However, modifying the nominal b-value leads to a different TE for each scan (see Eq. 8). This causes different signal dropout artifacts between the scans and potentially different eddy current distortion, making the alignment of the DW images challenging and perturbing the MFM estimation. Additionally, acquisition of several separate single-shell HARDI is more prone to patient motion between the scans. Multi-shell HARDI combines in a single acquisition the sampling of multiple spheres in q-space by modulation of 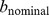 with various gradient strengths 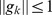. Because the unit-norm gradients 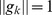 correspond to the shell of largest radius, this requires to set the nominal b-value based on the highest b-value of the acquisition. Since imaging of high b-values (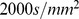 or more) is known to provide a better separation of multiple fascicles and to facilitate the estimation of their orientation [14], a multi-shell HARDI with a high nominal b-value should be preferred. This, however, results in a substantially increased TE (see Eq. 8). This results in an increased acquisition time and a lower signal-to-noise ratio for *all* the measurements (see Eq. 1), impacting both the low and the high b-value measurements. Additionally, imaging a higher nominal b-value is generally achieved by using longer diffusion gradient pulses, which in turn leads to larger eddy current distortion.

We propose instead the novel CUbe and SPhere (CUSP) acquisition technique. We incorporate multiple non-zero b-values by combining a single-shell HARDI acquisition at a specified 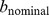 with gradients lying in the enclosing cube of the shell [67]. More precisely, the single-shell HARDI uniformly samples the diffusion signal on the hemisphere, which is described by unit-norm gradients 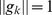. This shell employs the b-value providing the optimal SNR for the diffusion weighted acquisition. It can be determined by 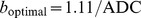 [10], [68], and is often suggested to be 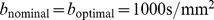 for an adult brain and 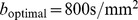 for a pediatric brain [69]. The single-shell HARDI provides a full spherical sampling with the optimal SNR and the optimal TE for the b-value 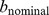. We then acquire additional b-values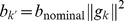 *without* modifying the TE by modulation of 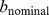 with gradients whose *strengths is greater than 1*: 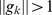. The only constraint for 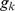 is to have unit norm components, corresponding to the normalized current intensity in each gradient coil. Denoting by 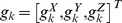 the gradient components, this leads to 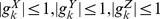 which describes the enclosing cube of the sphere of radius 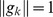. We call this region the *cube of constant TE*. Any gradient in this region can be acquired without modifying the TE by choosing the appropriate gradient strength. Because the diffusion sensitivity is dependent on the square of the gradient norm, imaging in the *cube of constant TE* enables the acquisition of b-values up to 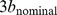. This maximum b-value is obtained when using the four non-symmetric 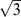-norm tetrahedral gradients [9] lying on the corners of the cube of constant TE (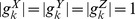).

**Figure 3:**
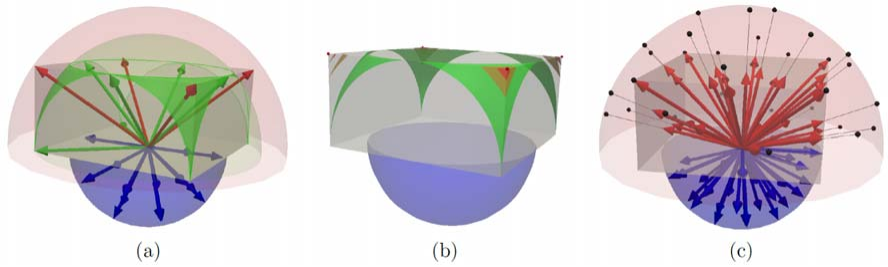
CUbe and SPhere (CUSP) imaging can be constructed as a truncated or a projected multi-shell HARDI. (a): In CUSP-T (Truncated), we consider a multi-shell HARDI with uniformly spaced radius (blue, green, red) and truncate those parts of the shells that project outside of the cube of constant TE of the inner shell. (b): CUSP-xT (eXponential Truncated) employs portion of multiple shells with exponentially spaced radius to achieve an improved uniformity of SNR. (c): In CUSP-P (Projected), we consider an inner shell at 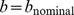 (blue) and an outer shell at 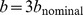 (red). The gradients of the outer shell are projected to the cube of constant TE (grey) to avoid any increase in TE. In these figures, the spherical and cubic sampling were shown in different partitions of q-space for visualization purpose.

We envisage three ways to construct a CUSP acquisition which are based on a generalization of a multi-shell HARDI (see Fig. 3). First, in CUSP-T (Truncated), we consider a conventional multi-shell HARDI composed of 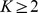 shells and truncate those parts of the shells that project outside the cube of constant TE of the inner shell (Fig. 3a). In this acquisition scheme, multiple shells with uniformly distributed b-values across the cube of constant TE are employed. However, the signal strength varies as 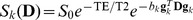 and so the SNR exponentially decreases with increasing b-value 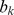. Therefore, in CUSP-xT (eXponential Truncated), we propose to employ shells with exponential spacing (Fig. 3b). The obtained exponentially increasing shell density with increasing b-value enables us to counter-balance the loss in SNR. This samples q-space in a manner that achieves an improved uniformity of SNR. Finally, in CUSP-P (Projected), we consider an acquisition composed of an inner shell at 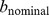 and an outer shell at 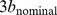. The outer shell passes exactly through the corners of the cube of constant TE. Any other gradients of this shell are outside of the cube and cannot be imaged without modifying the TE. Instead, we propose to project them onto the faces of the cube of constant TE (Fig. 3c) by reducing the gradient strength until the cube surface is reached. This preserves the gradient orientations and provides high angular resolution imaging with a large number of different b-values above 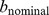 without any additional cost in TE. The gradient scheme optimization algorithm of Cook *et al.* [70] can be used to identify maximally isotropic subsets of gradient orientations between the shells. Furthermore, if desired certain gradient directions and strengths can be fixed and others optimized around them.

Our work is the first report of utilizing such a CUbe and SPhere acquisition to enable the full estimation of a multiple fascicle model. The strength of our technique is to provide multiple non-zero b-values and higher b-values than the nominal b-value *while* achieving the same low TE as a single-shell HARDI. Consequently, it does not increase the imaging time, does not increase the eddy current distortion and provides the optimal signal-to-noise ratio for all the measurements.

### 4 Summary

We provided the theoretical demonstration that only the tensor orientation can be uniquely estimated when using a single non-zero b-value. Multiple non-zero b-values are required to fully estimate the tensors' direction in addition to the tensors' size and their respective fraction of occupancy. We proposed a novel algorithm for the estimation of the parameters of a multi-fascicle model (MFM). It is formulated as a log-Euclidean *Maximum A Posteriori* estimation problem. It ensures we estimate non-degenerate tensors and incorporates a finite difference spatial regularization scheme. In conjunction with our optimization algorithm we provided an optimal CUbe+SPhere (CUSP) imaging strategy based on a generalization of a multi-shell HARDI. It satisfies the requirement of multiple non-zero b-values and incorporates high b-values *while* employing the same minimum achievable TE as a single-shell acquisition. Consequently, the imaging time and the eddy current distortion are not increased. Compared to a multi-shell HARDI, CUSP achieves a better SNR. The performance and properties of our novel CUSP-MFM technique are investigated through several experiments described in the next section.

## Methods

### MFM estimation algorithm

The multi-tensor estimation algorithm was implemented in C++ and parallelized over the image space. The model parameters were set as follows. The diffusivity of free water at 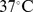 was set to 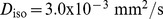 [17]. The anisotropic regularization parameter 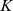 was set to 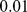 and the regularization influence parameter 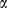 progressively increased between 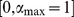, playing the role of the inverse of a decreasing temperature as proposed in [71]. This allows to first explore a larger number of solutions (high temperature) and in a second step to constrain the solution by gradually increasing the weight of the neighborhood (low temperature). Since the minimization was performed with the BOBYQA algorithm, which is a derivative-free optimization technique, we used the original 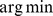 operator in Equation 6 and not its softmax approximation. Depending on our experiments, we considered a maximum of 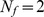 or 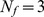 tensors per voxel. The isotropic water fraction was initialized to 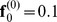 and the fascicle fractions to 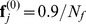 for 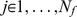. All parameters may be estimated simultaneously with CUSP-MFM. However, in order to reduce the number of parameters, each tensor was constrained to have a cylindrical shape by setting 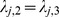 for 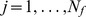. A cylindrical shape was also employed in [15] and is generally considered reasonable with regard to the expected shape of a fascicle. Consequently, fitting our model involved the estimation of 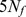 free parameters: four parameters per tensor, and 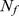 parameters for the 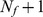 fractions. Model order selection was used to determine the number of fascicles at each voxel when appropriate. This was achieved by the F-test method [44]. A number of other model selection approaches have been investigated in the literature [43], [44], [61], [72]. Their comparison, however, fall outside of the scope of this current work.

### Two-tensor synthetic data

We generated various synthetic phantoms to evaluate CUSP-MFM. The tensor profile 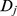 representing an individual fascicle was chosen to match typical in vivo data observations. A trace of 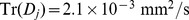 was chosen [12], and varying FA for each tensor (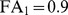; 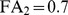) simulated. This was achieved by considering the following relationship between the eigen-values 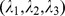 of a cylindrical tensor 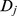 and the tensor FA and trace 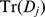: 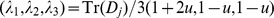 with 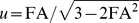. The fractions for the isotropic compartment (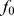) and the tensors (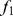, 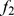) were set to 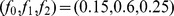. The diffusion-weighted signal was simulated for different acquisition schemes and corrupted by various Rician-noise levels. The reported SNR were computed on the 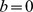 s/mm^2^ images.

We focused here on short duration acquisitions with a low number of directions which are of practical interest for clinical applications. We considered a CUSP-T acquisition consisting of a total of thirty-five images, referred to as CUSP35 (Fig. 3a). CUSP35 was constructed from a *truncated three-shells HARDI* composed of five 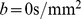, an inner shell of sixteen directions at 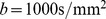, a second shell at 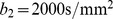 and a third shell at 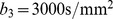. The gradients of the inner shell were uniformly distributed on the hemisphere by minimizing the sum of the electrostatic repulsive forces [10]. The second shell was truncated to the cube of constant TE by imaging the six hexahedral gradients which are located at the intersection of the second shell and the edges of the cube of constant TE. The truncation of the third shell to the cube of constant TE led to the four 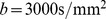 tetrahedral gradients only. Two repetitions of these gradients were achieved [67] to counterbalance the lower SNR associated with such high b-value measurements.

We compared the CUSP35 acquisition scheme to a single-shell HARDI acquisition, referred to as HARDI35, which includes five 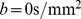 images and one shell of thirty directions at 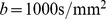. We also considered an acquisition scheme with five 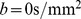 and 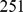 unique directions (HARDI256). Again, we employed the electrostatic repulsion algorithm of [10] to determine uniformly distributed gradient orientations on the hemisphere for both HARDI35 and HARDI256. In the following, HARDI35-MFM and CUSP35-MFM refers to the MFM estimation performed by our novel algorithm with respectively the HARDI35 and the CUSP35 acquisition schemes. Identical estimation parameters were employed in HARDI35-MFM and CUSP35-MFM.

For each experiment we reported both qualitative and quantitative results. For the quantitative analysis, we compared the estimated multi-fascicle model to the synthetic ground truth by means of different metrics. The tensors were compared in term of average log-Euclidean distance (tALED), taking into account a possible permutation between the estimated and the reference tensors:
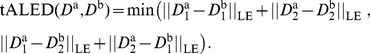
Using the log-Euclidean metric enables us to fully compare the tensors and not just the crossing angle as frequently done in the literature. The corresponding fractions were compared in terms of average absolute difference (fAAD). We also compared our multi-fascicle model to the ball and stick model [42] implemented in FSL. Since this model estimates only the fascicle orientations it was not possible to compare the full tensors nor to compare diffusion scalar parameters. We consequently compared our fitting algorithm to the ball-and-stick algorithm by assessing the average minimum angle (tAMA) [48] widely used in the literature. Finally, we simulated the diffusion signal arising from two uniform crossing fascicles, for various Rician noise corruption levels (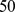
dB and 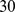
dB). We carried out the MFM estimation and then characterized the uniformity of diffusion scalar parameters along the fascicles.

### In vivo data

The performance of CUSP-MFM was assessed on in vivo data acquired on a Siemens 3T Trio scanner with a 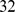 channel head coil. The scanned subjects were all healthy volunteers, of age between 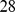 and 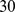 years old. The acquisition parameters used were as follows: 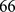 slices, FOV = 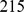
mm, matrix = 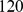
×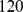, resolution = 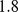
×
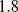
×
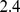
mm^3^. Eddy current distortion was minimized by utilizing a twice-refocused spin echo sequence [23]. In the first experiments, we employed the same gradient strength and orientation as those used in our synthetic experiments (CUSP35 and HARDI35). The minimum achievable TE/TR for both CUSP35 and HARDI35 were *identical* and equal to 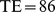
ms/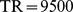
ms, achieving an acquisition duration lower than 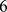 minutes.

We acquired a multi-shell HARDI composed of 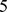 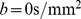 and three shells of 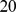 gradient directions each at 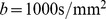, 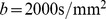 and 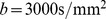, referred to as MSHARDI-65. Maximally isotropic gradient subsets were obtained by using the algorithm of [70].

We acquired a CUSP-P acquisition referred to as CUSP65 and constructed from a *projected two-shell HARDI* (Fig. 3c) consisting of 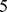 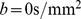 images, 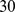 gradients on the inner shell at 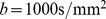 and 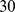 gradients on the outer shell at 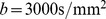.

We employed a generalization of the optimization algorithm of [70] to determine maximally isotropic gradient subsets for such a CUSP-P. More precisely, we first optimized the subset of 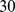 gradients of the inner shell with the electrostatic repulsion model of [10], providing uniformly distributed gradient directions on the hemisphere. We then optimized the second subset of 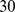 gradients with the electrostatic repulsion model of [10] while (1) taking into account the repulsion in orientation with the first subset and (2) enforcing the inclusion of gradients at 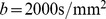 and 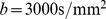 to ensure that high b-value images are acquired and for comparison to the multi-shell HARDI. The gradients of this second shell were projected to the cube of constant TE to avoid any increase in TE (see Fig. 3c) compared to imaging the inner shell only. The TE for MSHARDI-65 and CUSP-65 was respectively 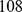
ms and 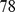
ms, and the acquisition time lower than 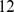 minutes.

Finally, a T1-weighted MPRAGE image was acquired with the following parameters: 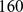 slices, FOV = 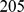
mm, matrix = 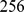
×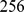, resolution = 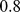
×
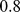
×
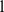
mm^3^, TE = 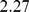
ms, TR = 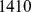
ms, 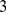
min 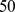
sec. This anatomical scan was used to visualize the results.

The diffusion weighted images were corrected for head motion during the scan by rigid registration of the DW-images to the 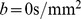 image [73]. The gradient orientations were compensated for the rotation component of the transformation for each image. We considered the estimation of 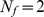 and 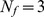 fascicles, with and without employing the F-test model selection. The multi-fascicle model estimation time was approximately 1 hour and 30 minutes for 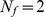 and 2 hours for 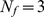 on a 8 Core 3 Ghz Intel Xeon. We compared the 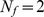 fascicles case with the ball-and-stick model implemented in FSL [42]. We also estimated the ball-and-stick model after noise correction of the DW images with the Joint Linear Minimum Mean Squared Error (LMMSE) filter proposed by [74].

We performed an experiment to examine the effect of CUSP-MFM on the assessment of tensor diffusion parameters. We applied 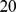 random rotations to both the in vivo CUSP35 and HARDI35 acquisitions. This simulates variations of the partial volume effect in each voxel, and consequently variations of the partial volume fractions of each tensor in each voxel. However, the fractional anisotropy should be stable across the rotations. We selected a fascicle of the corticospinal tract and assessed the fraction anisotropy along this same tract for the multiple rotations. We compared the results when using CUSP and the single-shell HARDI.

Finally, a *quantitative* comparison of CUSP to a multi-shell HARDI was achieved by assessing the estimation uncertainty via *residual bootstrapping* [75]. The residual bootstrap is a model-based resampling technique. It is based on the estimation of a model (here the multi-fascicle model) and on the generation of a set of virtual new DWI acquisitions by randomly sampling the model residuals. In contrast to repetition-based resampling techniques, it does not require any repetition of the gradient directions during the acquisition. Contrary to the wild bootstrap [76], it does not assume any symmetry in the distribution of the residuals. The residual bootstrap has been shown to lead to smaller biases and reduced overall errors in comparison to the wild bootstrap, enabling the estimation of uncertainties with higher accuracy [75]. Here, the residual bootstrap method was employed to quantitatively compare the estimation uncertainty with CUSP-65 and with MSHARDI-65.

## Results

### Synthetic data

**Figure 4:**
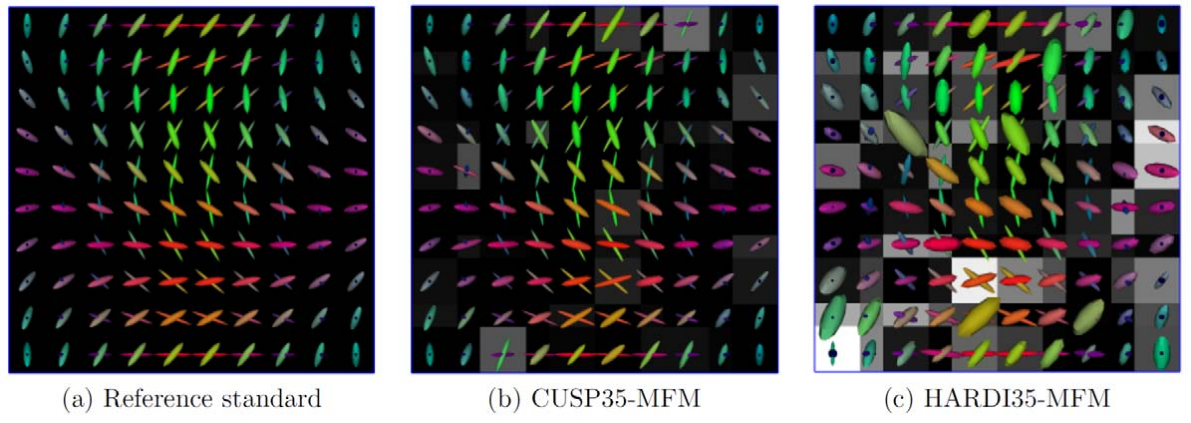
Qualitative evaluation of CUSP-MFM. (a) One hundred synthetic tensors crossing at 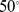 in various configurations. (b) Estimated tensors with a CUSP35 gradient encoding scheme (SNR = 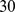
dB) superimposed on the first tensor's fraction 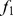 (window: 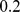 ; level 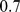). (c) Estimated tensors with the HARDI35 gradient encoding scheme. With a single non-zero b-value (Fig. c), the tensor eigenvalues and the fractions are collinear, leading to a poor multi-fascicle estimate. When using CUSP35 (Fig. b), the system is better determined, leading to a better estimate. Both the tensors and the fraction 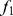 are more uniform when using CUSP35-MFM compared to HARDI35-MFM.

We generated a set of phantoms containing one hundred two-tensor models separated by a given angle *with various orientations* (see Fig. 4a). Ten phantoms with crossing angles from 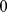 to 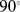 were generated. Fig. 4 qualitatively shows the improvement when estimating the tensors from the CUSP35 acquisition (Fig. 4b) compared to a single-shell HARDI35 acquisition (Fig. 4c). Both the tensors and the fraction 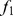 are more uniform with CUSP35-MFM.

**Figure 5:**
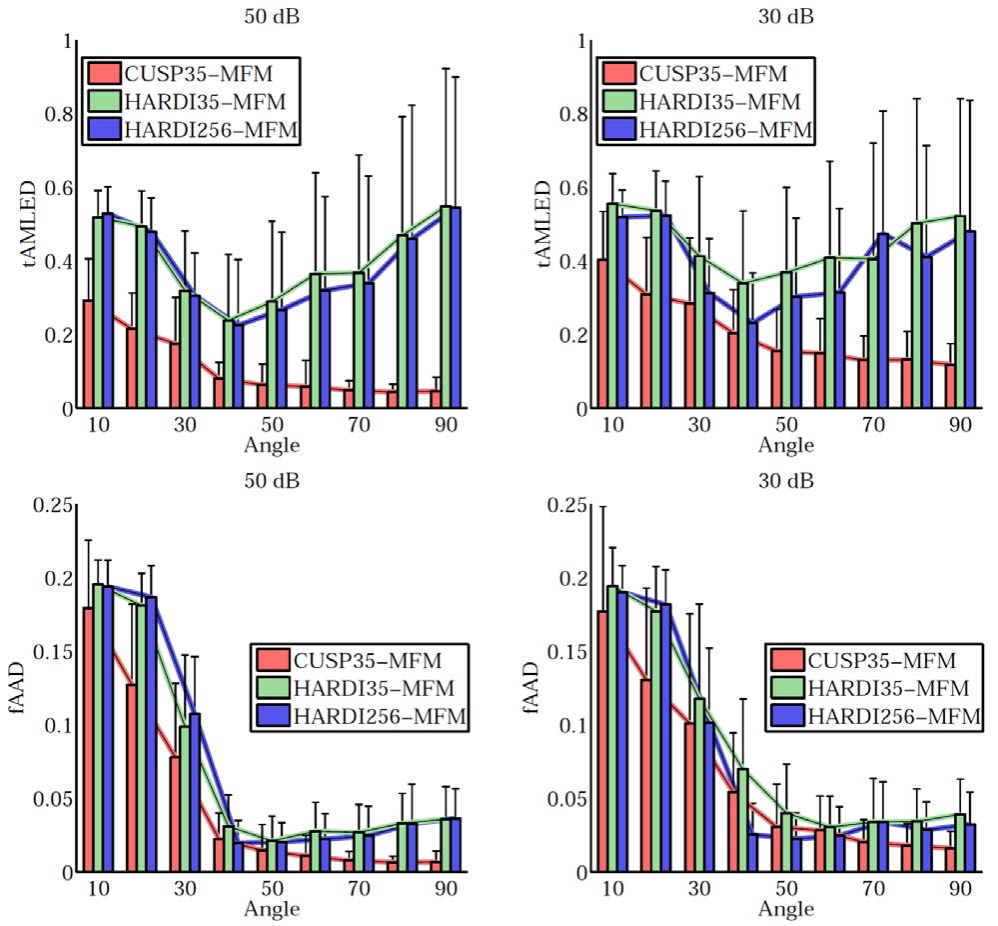
Quantitative evaluation of the CUSP-MFM estimation accuracy. Quantitative evaluation of the estimation accuracy for the fractions (first line, fAAD metric) and the tensors (second line, tALED metric). Each plot shows the quality metric (fAAD, tALED) in function of the crossing angle for various gradient encoding scheme and various signal-to-noise ratios. It shows that employing a large number of directions (HARDI256) does not dramatically improve the results whereas introducing multiple non-zero b-values does (CUSP35). CUSP35-MFM consistently provides the best estimation accuracy.

Fig. 5 quantitatively reports the estimation accuracy for various SNR (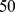
dB, 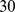
dB). It shows for each angle (from 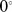 to 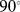) the mean and variance of the tALED and fAAD metrics over the one hundred tensors. Particularly, it shows the CUSP35 encoding scheme achieves better results than HARDI35 and HARDI256. It experimentally supports our theoretical demonstration that multiple non-zero b-values are required to fully estimate the tensors. Employing even up to 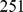 unique directions does not dramatically improve the results since it does not solve the collinearity of the parameters.

**Figure 6:**
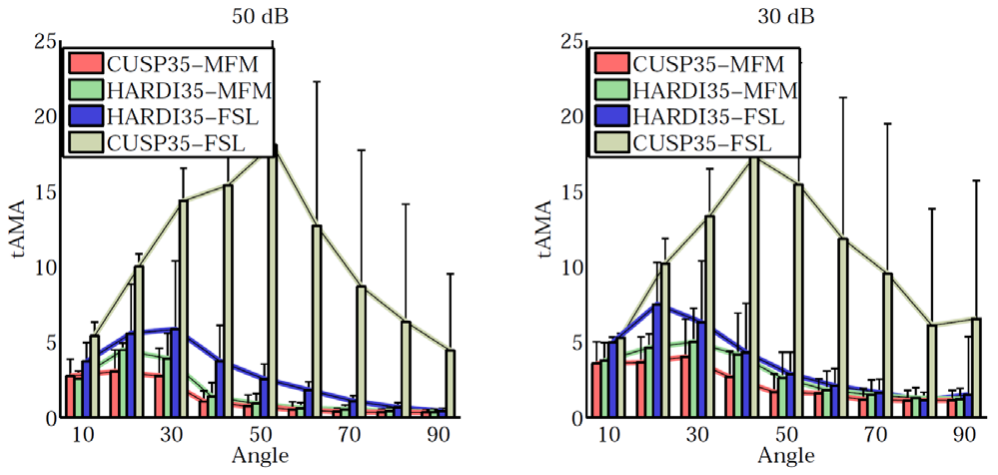
Quantitative evaluation of the angle detection accuracy. Evaluation of the angle detection accuracy in term of average minimum angle error (tAMA) and comparison with the ball-and-stick model of FSL. CUSP35-MFM provides on average the best angular resolution, particularly for angles lower than 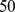 degrees, while it provides more information for clinical studies by estimating the full tensors: diffusion parameters such as the fractional anisotropy or the radial diffusivity can be computed for each fascicle independently.

Fig. 6 shows the comparison of our MFM algorithm to the the ball-and-stick algorithm implemented in FSL [42]. We noticed that FSL does not perform well with the CUSP35 acquisition scheme. CUSP35-MFM provides the best angular resolution compared to the other approaches, particularly for small angles, while it provides more information by estimating the full tensors. With CUSP-MFM, each fascicle can be characterized by evaluating the diffusion tensor parameters (FA, MD, etc.) of each tensor.

**Figure 7:**
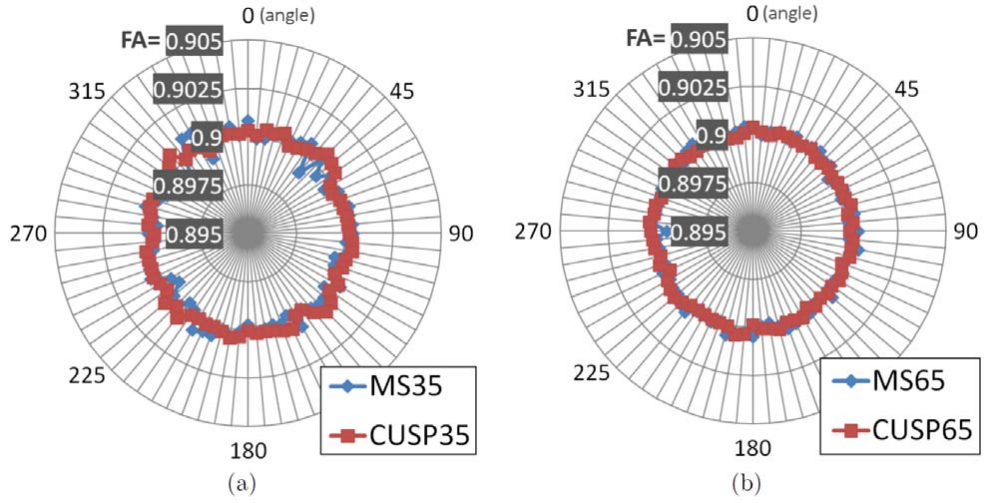
Angular dependency of the fractional anisotropy with CUSP and a multi-shell HARDI. The DW-images for a single tensor with constant FA (FA = 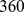) were simulated one hundred times for various tensor orientations (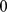
–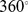) and for both CUSP and a multi-shell HARDI, corrupted by Rician noise (SNR on the 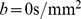: 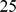
dB). We report the mean of the estimated FA for each angle, for CUSP-35 (a) and CUSP-65 (b). The average FA is compared to the average FA obtained when using MSSHELL-35 (MS35: 5 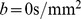 and three shells of 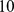 gradients each at 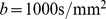, 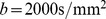, 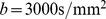) and MSSHELL-65 (MS65). The angular dependency of CUSP and of a multi-shell HARDI is similar.

Finally, we investigated whether or not CUSP introduces an angular preference for certain spatial directions when characterizing fascicles (Fig. 7). We simulated the DW-images for a single tensor with constant FA (FA = 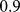) with various orientations, for both the CUSP and the multi-shell HARDI acquisitions. The tensor was rotated around its third eigenvector by increments of 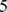 degrees between 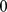 and 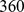 degrees. For each orientation, the tensor representing the fascicle was estimated, and its FA assessed. This was repeated one hundred times. Fig. 7 shows the mean FA for each tensor orientation over the hundred repetitions. The mean FA obtained with a multi-shell HARDI and with CUSP are comparable, showing that such a multi-shell HARDI and CUSP have a uniform angular sensitivity to fascicle orientation.

**Figure 8:**
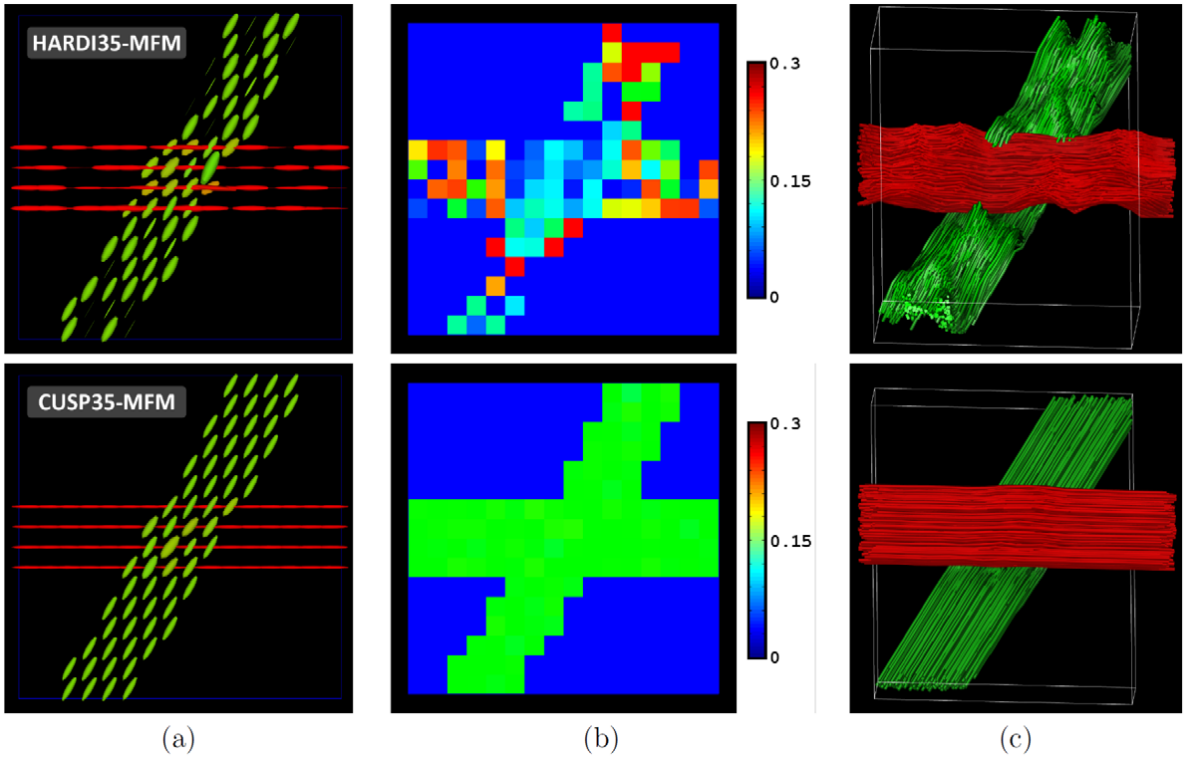
Two uniform crossing fascicles have uniform characteristics (FA) with CUSP-MFM. Estimation of two synthetic crossing fascicles (angle = 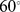, SNR = 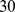
dB) with HARDI35-MFM (first line) and with CUSP35-MFM (second line). (a) Estimated tensors and (b) fraction of the isotropic water compartment. (c) Illustration of the fractional anisotropy uniformity for each fascicle. In this image, the z-coordinate of the tract streamlines encode for the fractional anisotropy along the tracts (red: 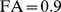; green: 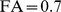). It shows the FA of two uniform fascicles to be qualitatively more uniform with CUSP35-MFM than with HARDI35-MFM.

We then generated a phantom representing two uniform fascicles (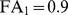 ; 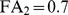) crossing with an angle of 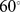 (see Fig. 8). In this experiment, the F-test model selection was used. The resulting multi-tensor field is reported in Fig. 7a. It shows more uniform tensors when using CUSP35-MFM. Fig. 7b reports the estimated fraction 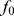 of the isotropic diffusion compartment. It shows that CUSP35-MFM provides an estimate of 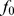 very close to the true simulated value (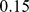), while the isotropic water fraction cannot be accurately estimated with HARDI35-MFM. Finally, Fig. 7c depicts the FA (non-)uniformity along the fascicles. For illustration, we modified the z-coordinate of the tract streamlines to encode for the FA along the tract. It shows that CUSP35-MFM provides a much more uniform FA than HARDI35-MFM.

**Figure 9:**
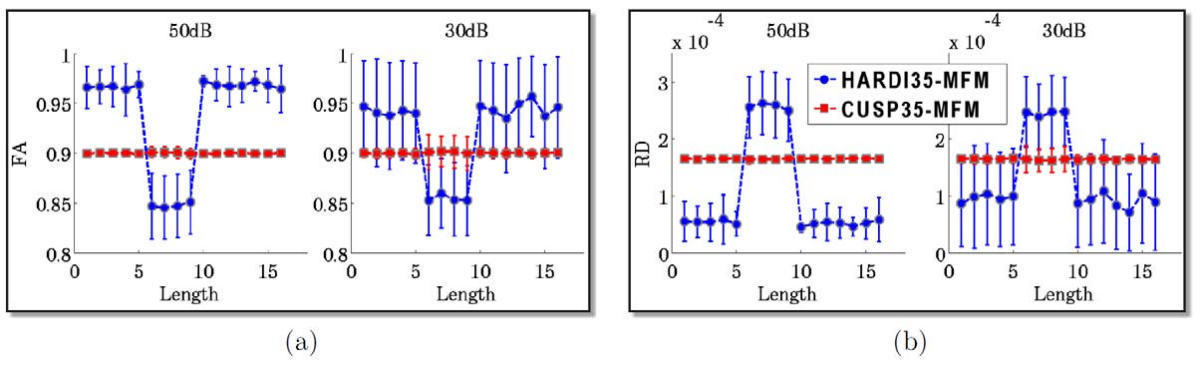
Quantitative evaluation of the fascicle characteristics along a uniform fascicle. Quantitative evaluation of diffusion parameters along the horizontal synthetic tract of Fig. 8 for SNR of 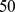
dB and 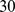
dB and for HARDI35-MFM and CUSP35-MFM. (a) Fractional anisotropy assessment. (b) Radial diffusivity assessment. It shows the FA and the RD of two uniform fascicles to be almost uniform with CUSP35-MFM, which is clinically relevant to assess individual fascicle characteristics when studying white matter development or degeneration.

These findings were quantitatively verified by simulating one hundred times the diffusion signal for different signal-to-noise ratios (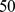
dB and 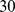
dB). Fig. 9 reports the mean and variance of the FA (Fig. 9a) and of the radial diffusivity (RD) (Fig. 9b) over these experiments. With CUSP35-MFM, the FA and the RD of two uniform fascicles is distinctly more uniform than with HARDI35-MFM, which is highly relevant to accurately characterize the fascicles.

### In vivo data

**Figure 10:**
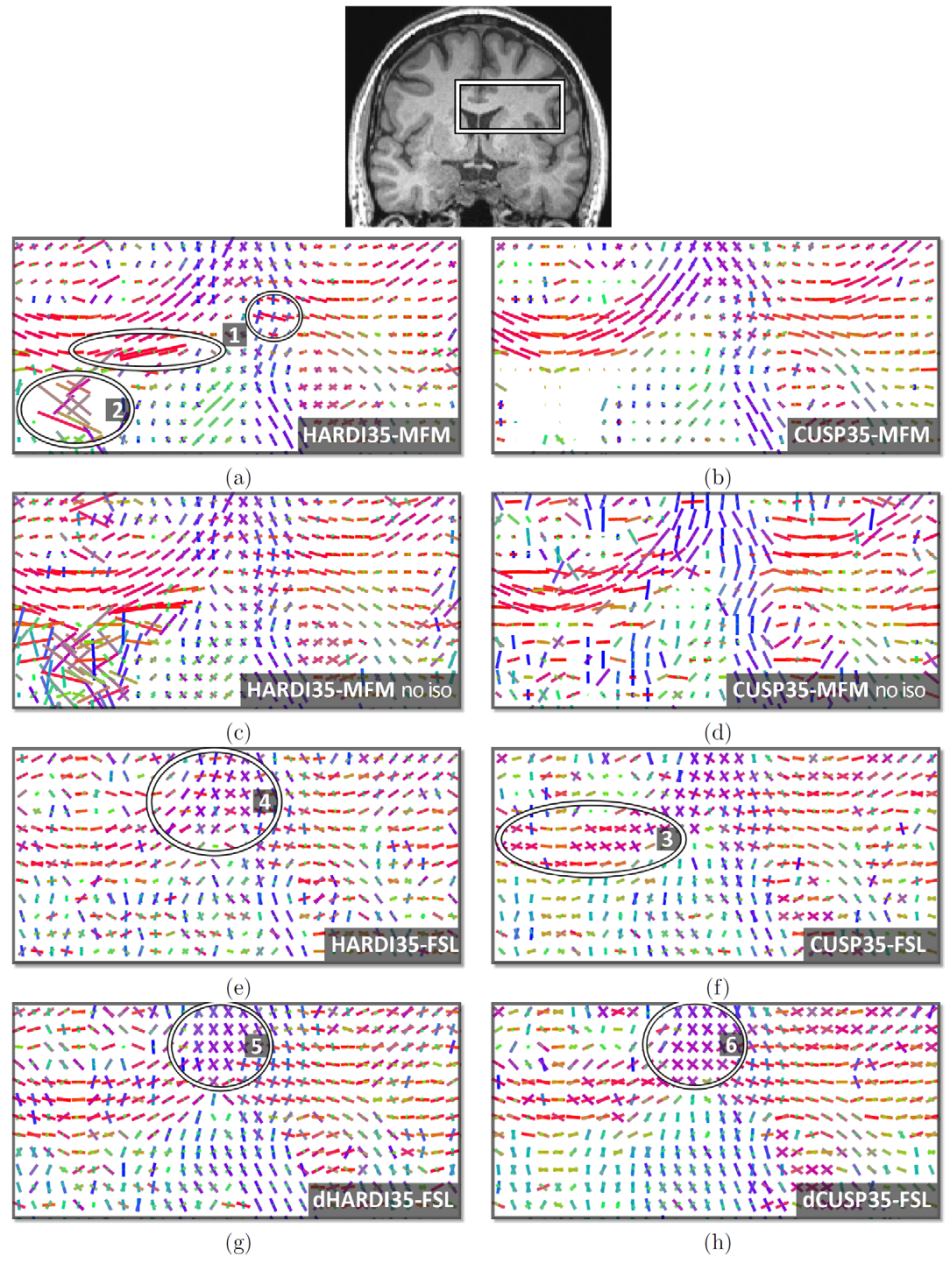
Evaluation with in vivo short duration DWI acquisitions with 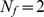, without any model selection. Comparison of the HARDI35 (first column) and CUSP35 (second column) acquisitions. Fig.a and Fig.b: in contrast to CUSP35-MFM (b), HARDI35-MFM (a) leads to degenerate tensors (area 1) and confounds CSF contamination and fascicles (area 2). Fig.c and Fig.d: when ignoring the estimation of the isotropic compartment, the performance of CUSP35-MFM (d) are strongly affected. The diffusion of unrestricted water cannot be ignored when using a multiple b-values acquisitions. Fig.e and Fig.f: FSL estimates sticks with noisy orientation (area 4), and leads to non-aligned sticks in a single fascicle region of the corpus callosum (area 3). Fig.g and Fig.h: FSL estimation after denoising the DW images (dHARDI35 and dCUSP35).

We report in this section the results of experiments on in vivo data. In Fig. 10, we qualitatively compare the multi-tensor estimation performances when using a HARDI35 acquisition scheme (first column) and a CUSP35 acquisition scheme (second column). In this experiment, to fully characterize our multi-fascicle model approach, we did not employ any *model order selection* but estimated 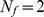 tensors at each voxel. Tensors with estimated fraction of occupancy lower than 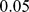 were, however, not visualized. In the first line we compare the results of HARDI35-MFM (Fig. 10a) and CUSP35-MFM (Fig. 10b). HARDI35-MFM leads to tensors with degenerate tensor size (areas 1), and leads to non-null tensors in the CSF (area 2), confounding isotropic water fraction and mixture of fascicles. In contrast, CUSP35-MFM achieves a better tensor uniformity.

Fig. 10c and Fig. 10d reports the results of HARDI35-MFM and CUSP35-MFM but *without* the estimation of the isotropic compartment. It shows that ignoring the isotropic compartment has substantially more impact when using CUSP35 (see Fig. 10d). Fig. 10e and Fig. 10f reports the results of the ball and stick model of FSL. It results in distinct estimated sticks in the body of the corpus callosum (area 3), a region known to contain a single fascicle orientation, probably fitting the noise. Additionally, sticks with orientations matching poorly the known anatomy are estimated (area 4). Thirty-five directions is perhaps not enough for this MCMC Bayesian ball-and-stick model to be accurately estimated. Applying a preprocessing noise-correction filter (Fig. 10g and Fig. 10h) improves the results but creates anatomically implausible crossings (areas 5 and 6).

**Figure 11:**
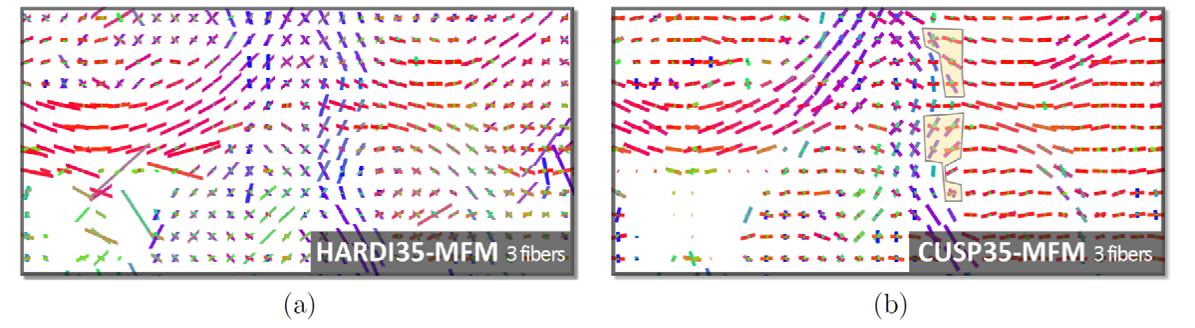
Evaluation with in vivo short duration DWI acquisitions with 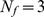 fascicles, without any model selection. Estimation of 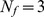 fascicles with HARDI35 (a) and with CUSP35 (b), which contain only 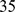 images. The tensors with fraction of occupancy smaller than 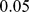 were not visualized. CUSP35-MFM results in the estimation of three fascicles in a region (see outlined voxels) that matches the known anatomy, the centrum semiovale.

Fig. 11 reports, for the same coronal slice, the MFM estimation results with 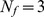 fascicles at each voxel *without* model order selection. Again, HARDI35-MFM leads to tensors with a degenerate size, and confounds the estimation of the isotropic water fraction and of the fascicles. In contrast, with CUSP35-MFM, the location of voxels in which three distinct fascicles are estimated with non-null fractions matches the known anatomy, whereas only 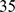 images were acquired. Additionally, the estimated orientation in the body of the corpus callosum matches the anatomy, whereas 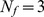 tensors were estimated at each voxel.

**Figure 12:**
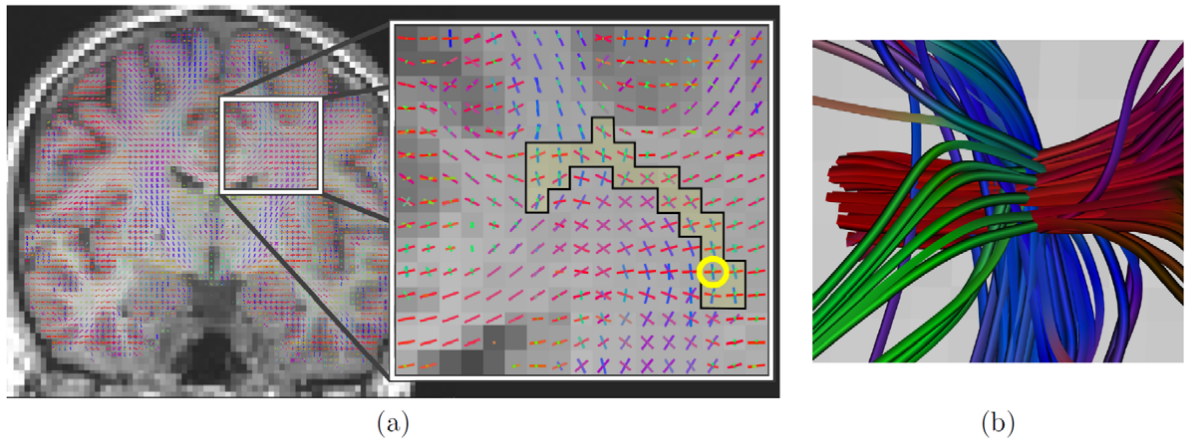
Estimation of 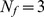 fascicles with F-test model order selection and CUSP-65. (a) Estimated MFM superimposed on the T1-weighted anatomical image. Particularly, three tensor were correctly estimated in the centrum semiovale, which is a known brain region in which three fascicles are crossing. (b) Illustration of the tractography streamlines passing through the voxel encircled in yellow in (a), showing the three crossing fascicles.

In Fig. 12, we show the CUSP-MFM estimation results when using the CUSP-65 acquisition and the F-test model order selection with a maximum of 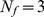 fascicles. Again, it shows estimated tensors that match the anatomy. Particularly, the outlined three tensor models correspond to a known region in the centrum semiovale where three fascicles are crossing.

**Figure 13:**
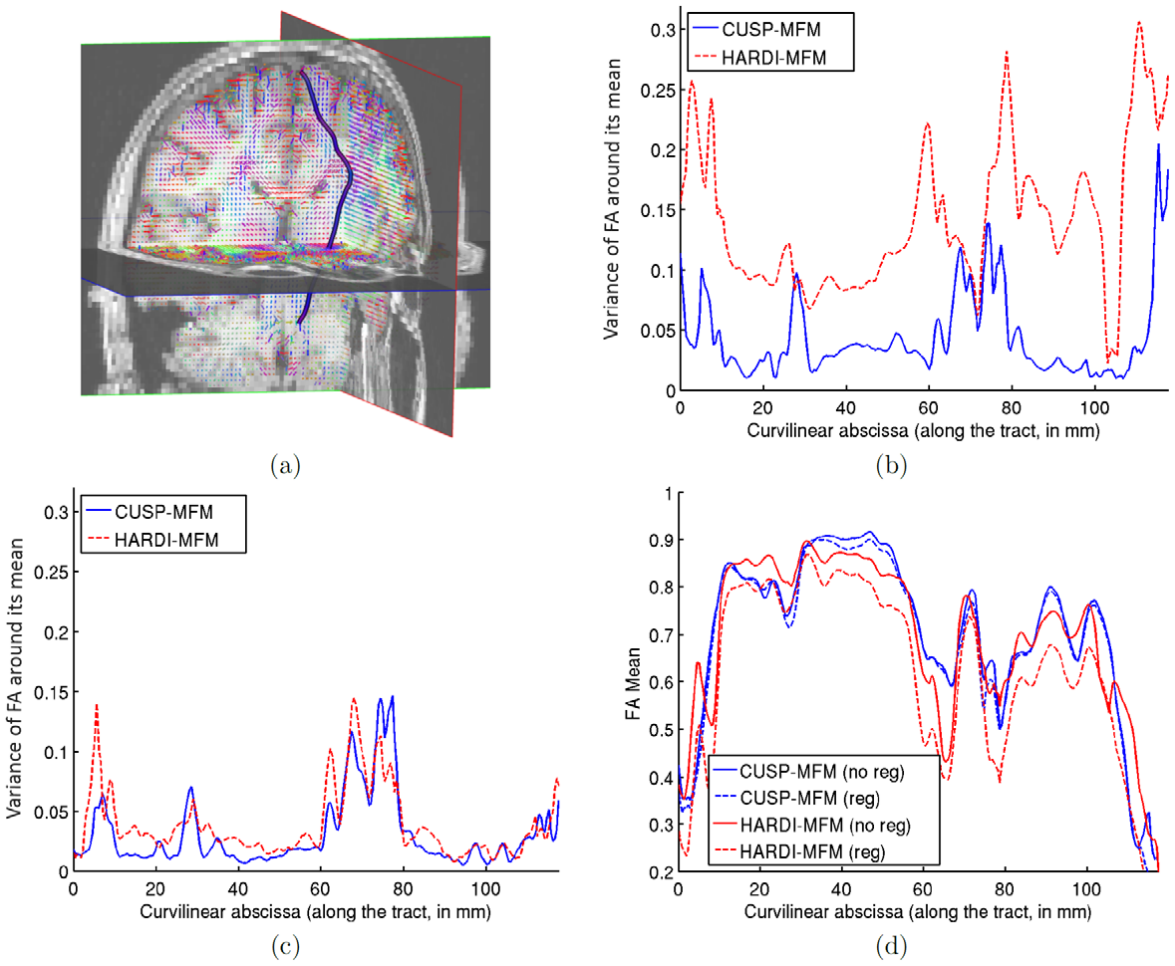
CUSP-MFM enables the estimation of diffusion tensor parameters which do not vary with the partial volume effect. We computed the FA along a same tract (Fig.a) for various artificial rotations of the diffusion-weighted images. For each streamline point, the most aligned anisotropic tensor with the streamline orientation was selected and its FA assessed. Fig.b shows the variance of the FA along the tract across the rotations, when using the CUSP or the HARDI acquisition and the MFM estimator without regularization and with the same parameters. HARDI has dramatically increased variance, as it conflates tensor size with partial voluming. CUSP does not. Fig.c shows the FA variance when adding the regularization to the estimation with both CUSP and HARDI. Fig.d shows the corresponding value of the FA along the tract. It shows that CUSP-MFM enables estimation of diffusion tensor parameters which do not vary with the partial volume fractions nor the regularization.

Fig. 13 demonstrates how CUSP-MFM enables the estimation of the *full* multi-tensor, including the fractions of occupancy of each tensor. We performed various rotations of the diffusion-weighted images to simulate various partial volume effects. We considered a reference tract belonging to the corticospinal tract (see Fig. 13a), applied the same rotation to each tract point, and assessed the fractional anisotropy *along the tract* across the rotations. Fig. 13b shows the variance of the FA when the MFM is estimated without any regularization, with CUSP35 and with HARDI35. It shows that the FA variance is much larger with HARDI35, because using an acquisition with a single non-zero b-value does not enable the full multi-tensor estimation. In contrast, the FA variance is reduced with CUSP35. Fig. 13c shows that when adding the regularization, the FA variance is reduced for both HARDI35 and CUSP35. However, as shown on Fig. 13d, adding the regularization has a significant impact on the mean of the FA with HARDI35, but not with CUSP35. With HARDI35, the regularization better constrains the optimization but leads to a wrong solution. In contrast, CUSP-MFM enables estimation of the full multi-tensor model, and consequently estimation of diffusion tensor parameters which do not vary with the partial voluming nor the regularization.

**Figure 14:**
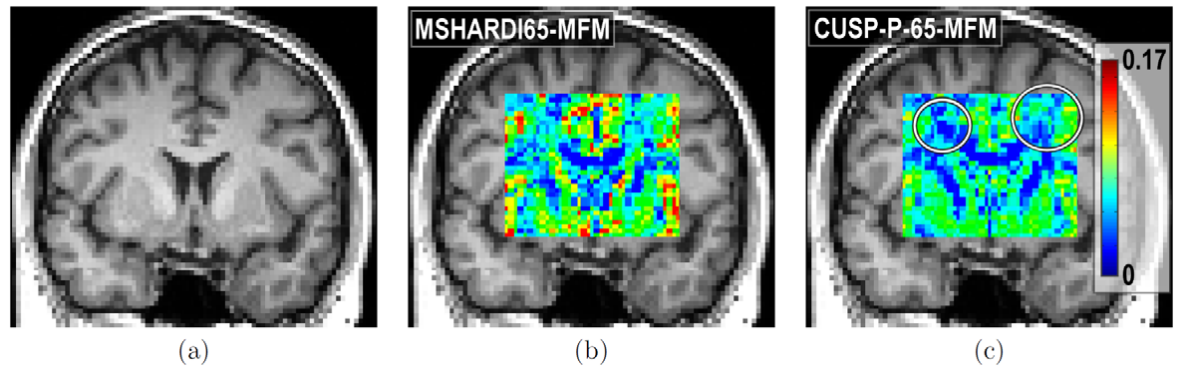
Comparison of CUSP and multi-shell HARDI via residual bootstrapping. (a) T1-weighted image showing the anatomy. (b) Standard deviation of the maximum FA when using MSHARDI-65-MFM. (c) Standard deviation of the maximum FA when using CUSP-65-MFM. The standard deviation of the maximum FA is significantly lower when using CUSP, showing a lower uncertainty in the MFM estimates.

Finally, we report in Fig. 14 the results of the residual bootstrap analysis, illustrating the benefits of employing CUSP instead of a multi-shell HARDI. From the initially fitted multi-fascicle model, we generated five hundred new virtual acquisitions. For each virtual acquisition, we estimated at each voxel the MFM and computed the maximum FA of the estimated fascicles. The maximum FA was used as a proxy to identify the same fascicle across multiple iterations of the bootstrap analysis. Fig. 14a and Fig. 14b shows the variance of the FA of the fascicle with the largest FA over the five hundred bootstrap iterations, respectively for MSHARDI-65-MFM and CUSP-65-MFM. It shows the uncertainty when estimating the fascicle of higher FA at each voxel. The uncertainty in FA is significantly lower with CUSP compared to the multi-shell HARDI, because MSHARDI-65 requires a larger TE to achieve a *nominal* b-value of 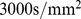 which leads to an acquisition with lower SNR.

## Discussion

Several methods have been investigated to overcome the limitations of DTI and to represent multiple white matter fascicles from diffusion-weighted imaging. Approaches such as DSI, QBI, EQBI, DOT, SD or GDTI focus on estimating the global shape of the diffusion profile resulting from multiple fascicles present in each voxel. The major drawback is they do not consider each fascicle independently. Consequently, they do not enable characterization of each fascicle, and do not enable comparison of the fascicle characteristics between individuals. The assessment of parameters such as the generalized fractional anisotropy (GFA) or the generalized mean diffusivity (GMD) has also been proposed [41]. However, these measures do not represent a fascicle property but a *dispersion property* of the diffusion signal inside each voxel. For example, a synthetic uniform fascicle can be simulated by considering an identical tensor at each voxel. Such a uniform fascicle crossed by another synthetic fascicle has a GFA that varies in the crossing region [41] because the dispersion of the diffusion signal is different in the crossing region. Therefore, the GFA which represents a voxel property provides misleading data. A uniform fascicle should have constant diffusion parameters along its path. This essential limitation reduces the scope of DSI, QBI, EQBI, DOT, or GDTI to connectivity analysis. In addition, these approaches generally require a relatively high number of DWI acquisitions, limiting their use in clinical practice. Recently, Raffelt *et al.* [77] have demonstrated through Monte Carlo simulations of a model of diffusion in cylinders of a certain size range with certain permeability characteristics, that the signal measured by a single shell HARDI acquired at 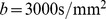 (with timing parameters achievable on a clinical MRI scanner) arises primarily from restricted water. Under these assumptions, the amount of signal can be related to the underlying density of white matter fascicles, thus enabling the formation of a measure of the ‘apparent fiber density’. In addition, the orientation of white matter fascicles can be determined from the local signal maxima.

In contrast, our model enables the determination of the orientation of the white matter fascicles, measures of their local diffusion properties and the characterization of an unrestricted water component that is important in assessing edema and inflammation. Multi-fascicle approaches generally require the determination of the number of white matter fascicles at each voxel. This and only this enables characterization of each fascicle in addition to the orientation information, which is of central interest to study the white matter development or degeneration in research and clinical practice. Recently [78], have proposed to estimate a general ODF at each voxel and then fit tensors on the ODF to extract geometric features of the peaks. However, this requires the estimation of the ODF, which is sensitive to noise and requires a high number of DW images. In contrast, direct estimation of a MFM from the DW images enables representation of multiple fascicles and involves a small number of parameters, requiring potentially a small number of acquisitions. MFMs allow the computation of diffusion parameters such as the FA for each fascicle, which is essential for straightforward characterization of multiple fascicles.

Multi-tensor models have however frequently been reported to be numerically challenging and unstable [43], [44], [48]. Among others, Kreher *et al.* [44] have observed that with a model including one entirely isotropic diffusion tensor and multiple entirely anisotropic tensors, the mean diffusion and the relative ratio of components could not be separated with DW images measured at a single high b-value. In this paper we have shown that the reason lies in a collinearity in the parameters when a single non-zero 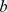-value acquisition is employed, leading to an infinite number of solutions. With only one non-zero b-value, as often used in the literature [19], [45], [48]–[50], the *tensor size* indicated by the magnitude of its eigenvalues and the estimated *volume fractions* are *collinear*. Consequently, fitting them together may make a fascicle with a uniform 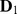 across its entire length grow and shrink as it passes through voxels and experiences different partial volume effects. Algebraically one constraint in the modeling could resolve this ambiguity: imposing a symmetry between the tensors' eigenvalues such as 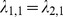 as used in [17]. Such a constraint is however not suited to accurately characterize each fascicle independently. In [44], [52], the fractions of occupancy were merged together. This, however, does not adequately represent the signal arising from each fascicle in presence of partial volume effect, and does not allow the computation of a full diffusion tensor associated with each white matter fascicle. Only the use of multiple non-zero b-values is a satisfying solution to disambiguate the estimation of the tensors and the fractions, and to enable characterization of each individual fascicle and of the unrestricted water component.

Our solution lies in a novel multi-fascicle estimation framework, CUSP-MFM, which is the combination of a novel multi-tensor estimation algorithm and an optimal acquisition scheme which satisfies the need of multiple non-zero b-values. The characteristics of our multi-tensor estimation procedure were driven by the objective to make it possible to represent multiple fascicles in clinical DWI with a relatively short acquisition time, compatible with pediatric and adult imaging. The *Maximum A Posteriori* formulation we employed enabled us to perform accurate and reliable multi-fascicle model estimation. Our model includes the estimation of an isotropic tensor which represents the free population of water molecules that is not affected by fiber structure barriers [42], [43], [45]. In this work we qualitatively did not observe a dramatic impact of adding this parameter when using a single-shell HARDI (Fig 10a and Fig 10c). In this case, there is no unique solution to the estimation due to the collinearity of the parameters, and the part of the DW signal coming from the free water population is incorporated in the tensor magnitude and in the fractions. In contrast, with multiple non-zero b-values (CUSP), the tensor magnitude and the fractions are not collinear anymore. We showed that the attempt to fit a model without the isotropic water compartment, *i.e.* a model that poorly represents the signal, perturbs the whole tensor estimation (Fig 10d). Therefore, to ensure reliable estimates, both multiple non-zero b-values and a model that accounts for the unrestricted water diffusion are necessary.

We show that when using an optimized acquisition scheme and when estimating the diffusion of unrestricted water, we can accurately estimate the fascicle orientation from the hindered diffusion. In this work, we have relied on the assumption that, *for each fascicle*, the DW signal mono-exponentially decays with increasing 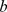-value. Based on non-monoexponential decays measurements in voxels, other authors have suggested that a non-monoexponential model may be more appropriate [55], [58]–[60], even for relatively low b-values [60]. However, these approaches have ignored the fascicle orientation heterogeneity (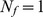) and the CSF contamination when considering a model with non-monoexponential decay. In this case, the source of the non-monoexponential decay remains unclear. As illustrated in Fig. 2, mixing of monoexponential decays as modeled in our approach does lead to a non-monoexponential behavior in voxels. As established by [16], when the gradient strength is limited and when the DW signal is properly modeled by taking into account both the fascicle orientation heterogeneity (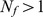) and the CSF contamination, a monoexponential decay for the signal arising from *each* fascicle can be safely assumed. In contrast, if the diffusion signal of a single fascicle exhibits a non-monoexponential decay, then our current multi-fascicle model cannot fully represent it and a generalization of our model may be necessary. Other per-fascicle models could be easily introduced in our framework and will be investigated in future work.

We have formulated our multi-tensor estimation approach in the log-Euclidean framework [65] which is an elegant way to guarantee that each tensor matrix is symmetric positive definite during the fitting. Other authors have considered a Cholesky parameterization of the diffusion tensor [79], or Bayesian approaches with priors on the eigenvalues [42], [45] to ensure valid tensors. With the log-Euclidean framework, all computations are performed within the appropriate manifold, ensuring valid tensors at each step. To our knowledge the log-Euclidean framework has never been employed for multi-fascicle models. Not only does it ensure non-degenerate tensors, it also provides us with a metric to compare and regularize tensors. All of the information carried by tensors is taken into account with the log-Euclidean metric, and not only features extracted from the tensors. Importantly, tensor determinants in this framework are monotonically interpolated. It prevents the regularized tensors from experiencing the *swelling* effect which has been observed in both the Euclidean and Cholesky frameworks [64], [65]. The swelling effect makes the estimated tensors larger than they should be. It particularly affects tensors at tissue borders such as the cerebrospinal fluid/white matter interface, where neighboring tensors are very dissimilar. Based on Monte Carlo experiments [80], have however suggested that an affine-invariant metric such as the log-Euclidean metric might lead to a larger bias in the case of extreme ADC values (either low or high). However, for the major brain tissues [80], shows that this bias is not observed.

We proposed a log-Euclidean regularization scheme which is not the direct extension of the one-tensor regularization. We suggested a particular approximation of the spatial gradient for multi-tensor fields (Equation 6). It relates neighboring tensors which are part of the same fascicle. Consequently, only tensors which are part of the same fascicle are regularized together. Introducing this regularization strategy is possible because we consider each fascicle independently. It would be difficult to integrate in DSI, QBI, GDTI, or SD because these approaches consider the general shape of the diffusion profile and not individual fascicles.

In this work we propose an acquisition scheme designed to enable accurate assessment of multiple white matter fascicles. Our CUSP (CUbe and SPhere) imaging technique combines a single-shell HARDI with gradients in its enclosing cube. The single-shell HARDI provides a full spherical sampling with the optimal SNR and the optimal TE for the chosen nominal b-value. Any gradient in the enclosing cube of the single-shell HARDI can be acquired *without* modifying the TE by choosing the appropriate gradient strength. It corresponds to a *cube of constant TE*. It enables acquisition of b-values up to 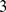 times the nominal b-value *while* achieving the same low TE as a single-shell HARDI. Consequently, and in contrast to multi-shell HARDI, it does not increase the imaging time, does not increase the eddy current distortion and it maintains the DWI signal-to-noise ratio by maintaining 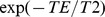. We envisaged three different CUSP variants (Fig. 3). The first, CUSP-T, is a truncated multi-shell HARDI. It employs the portion of multiple shells with uniformly spaced radius contained in the cube of constant TE. The second, CUSP-xT, is a truncated multi-shell HARDI which employs portions of shells with exponentially spaced radius to counter-balance the exponentially decreasing SNR with increasing b-values, and to achieve an improve uniformity of SNR. Finally, CUSP-P is a projected multi-shell HARDI, built by projecting the gradients of an outer shell at 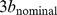 onto the faces of the cube of constant TE to avoid any increase in TE. This provides a uniform angular resolution and a large number of different b-values.

Our evaluation shows clear evidence that the estimation of both the tensors and the fractions of occupancy are improved when using CUSP instead of a single shell acquisition (Fig. 4, 5, 8). Additionally, we observed a substantial improvement of the angular resolution when using CUSP instead of a single shell (Fig. 6). From an algebraic point of view, only the tensor magnitude and the fractions are collinear with a single non-zero b-value. Introducing several non-zero b-values should not impact the tensor eigen vectors in a noise free model system. However, consistent with the literature [14], [43], [61], we observed that introducing higher b-values helps in differentiating the different compartments. Consequently, CUSP benefits are three-fold: First it solves the collinearity inherent to the multi-fascicle modeling with a single-shell HARDI. Second it enables imaging at higher b-values, which facilitates the estimation of the orientation of each fascicle [14]. Third, when compared to a conventional single-shell HARDI acquisition, it does not increase the acquisition time, does not increase the eddy current distortion and does not alter the signal-to-noise ratio.

The CUSP-MFM's performance was assessed via various experiments on both synthetic and in vivo data. We focused on short acquisitions suitable for routine clinical use, especially for pediatric MRI. The angular resolution is substantially superior to the state-of-the-art ball-and-stick model implemented in FSL (Fig. 6), while CUSP-MFM estimates the full tensor. This might be explained by the fact that the stick is an over-simplification which does not fully capture the true signal arising from a fascicle. Incorrect estimation of the tensor eigenvalues perturbs the estimation of the orientation because the optimization attempts at fully explaining the DW signal with an unrealistic model, leading to less accurate estimates. This also leads to the detection of multiple sticks in the body of the corpus callosum, which is a known single fascicle region (Fig. 10). In contrast, CUSP-MFM incorporates a more complex model for each fascicle and enables the assessment of individual fascicles characteristics in addition to the brain connectivity, by computing diffusion tensor parameter for each fascicle. The estimated diffusion parameters of two uniform synthetic crossing fascicles were shown to be almost uniform with CUSP-MFM (Fig. 8 and Fig. 9). It was verified on real acquisitions by simulating variations of the partial volume fractions for each tensor, by rotation of the diffusion-weighted images with various angles. We showed that with CUSP-MFM, the fractional anisotropy computed along a tract did not vary with the partial voluming effect nor the regularization, which was not the case when using a single-shell HARDI acquisition (Fig. 13). CUSP-MFM enables the full estimation of the multi-fascicle model, which enables characterization of the fascicles.

Fig. 5 showed that for small angles and in the absence of model order selection, the reconstruction error for the fractions of occupancy (fAAD) is significantly increased. Indeed, in the case where 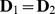, we have: 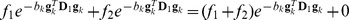. It is not possible to estimate the fractions of occupancy 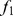 and 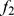 because of a collinearity in the parameters. The DW signal can be explained either with two compartments with non-null fractions 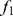 and 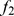 or with a single compartment with fraction (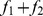). This case (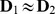), however, can be easily detected and handled with model order selection.

The qualitative evaluation on real data (Fig. 10, 11 and 12) showed that the estimated tensors orientation matches the expected underlying anatomy. The estimation without model order selection (Fig. 10, 11) showed that CUSP-MFM recovers the fascicle orientations better than the ball-and-stick model in regions of a single fascicle, either with 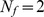 or 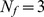. Importantly, the estimation of 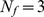 fascicles with only 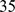 DW-images were consistent with the anatomy (Fig. 11). We also observed that in contrast to HARDI-MFM, CUSP-MFM enables correct estimation of the isotropic water fraction in the CSF (Fig. 10, 11).

Finally, we demonstrated that the estimation uncertainty is higher when using a multi-shell HARDI instead of CUSP (Fig. 14). This is due to the larger minimum achievable TE when imaging the full multi-shell HARDI, leading to an acquisition with exponentially decreased SNR (see Eq 1 and Fig. 1).

### Future work

Future work will focus on assessing different gradient schemes for the CUSP acquisition. Particularly, we will investigate the optimal ordering of the gradient directions. CUSP-MFM performance will be compared to Q-Ball Imaging and Spherical Deconvolution approaches for the assessment of connectivity.

Robust estimation will be also explored. It enables to reduce the influence of large residuals, making the estimation less sensitive to outliers than when using the least square criteria. It may provide a better robustness to patient motion and will be of particular interest for pediatric imaging.

## Conclusions

We demonstrated and experimentally verified that multiple non-zero b-values are required to fully estimate multi-tensor models. As a solution we proposed CUSP-MFM which combines an optimal CUbe and SPhere (CUSP) acquisition technique with a novel algorithm for the estimation of the parameters of a Multi-Fascicle Model (MFM). Our proposed CUSP acquisition technique provides multiple high b-values with the optimal achievable TE. It does not increase the imaging time nor the eddy current distortion compared to a single-shell HARDI. Additionally, it does provide the optimal signal-to-noise ratio, leading to estimates with higher certainty. Our novel multi-fascicle fitting algorithm MFM is formulated as a *Maximum A Posteriori* estimation problem. It integrates an isotropic compartment, constrained estimation and an original regularization scheme in which only the tensors that are part of the same tract are regularized together. It ensures non-degenerate tensors and robust-to-noise estimates. Our evaluation shows that CUSP-MFM enables the representation of multiple white matter fascicles from a short duration acquisition. It enables characterization of each fascicle, in addition to the brain connectivity, which is of great interest for clinical applications. CUSP-MFM may enable new investigations of the white matter development and degeneration in research and in clinical practice.

### CUSP Gradient Encoding Scheme

We provide the CUSP65 gradient encoding schemes in the Siemens format. On a Siemens scanner, this requires to set the imaged b-value to 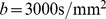 which corresponds to the b-value of the gradient of higher norm in the table. With this choice, the resulting minimum achievable TE correctly matches the minimum achievable TE of a single-shell HARDI at 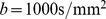.

[directions = 65]

CoordinateSystem = xyz

Normalisation = none

Vector[0] = (0, 0, 0)

Vector[1] = (0, 0, 0)

Vector[2] = (0, 0, 0)

Vector[3] = (0, 0, 0)

Vector[4] = (0, 0, 0)

Vector[5] = (1.00000, 0.00000, 0.00000)

Vector[6] = (0.16600, 0.98600, 0.00000)

Vector[7] = (0.11000, −0.66400, −0.74000)

Vector[8] = (0.90100, −0.41900, −0.11000)

Vector[9] = (0.16900, 0.60100, −0.78100)

Vector[10] = (0.81500, 0.38600, −0.43300)

Vector[11] = (−0.65600, −0.36600, −0.66000)

Vector[12] = (−0.58200, −0.80000, −0.14300)

Vector[13] = (−0.90000, −0.25900, −0.35000)

Vector[14] = (−0.69300, 0.69800, -0.17800)

Vector[15] = (0.35700, −0.92400, −0.14000)

Vector[16] = (0.54300, −0.48800, −0.68300)

Vector[17] = (0.52500, 0.39600, −0.75300)

Vector[18] = (0.63900, −0.68900, −0.34100)

Vector[19] = (−0.33000, −0.01300, −0.94400)

Vector[20] = (0.52400, 0.78300, −0.33500)

Vector[21] = (0.60900, −0.06500, −0.79100)

Vector[22] = (0.22000, −0.23300, −0.94700)

Vector[23] = (−0.00400, −0.91000, −0.41500)

Vector[24] = (−0.51100, 0.62700, −0.58900)

Vector[25] = (−0.41400, −0.73700, −0.53500)

Vector[26] = (−0.67900, 0.13900, −0.72100)

Vector[27] = (−0.88400, 0.29600, −0.36200)

Vector[28] = (−0.26200, −0.43200, −0.86300)

Vector[29] = (0.08800, 0.18500, −0.97900)

Vector[30] = (−0.29400, 0.90700, −0.30200)

Vector[31] = (0.88700, −0.08900, −0.45300)

Vector[32] = (−0.25700, 0.44300, −0.85900)

Vector[33] = (0.08600, 0.86700, −0.49100)

Vector[34] = (0.86300, 0.50400, −0.02500)

Vector[35] = (−1.00000, −1.00000, −1.00000)

Vector[36] = (1.00000, −1.00000, −1.00000)

Vector[37] = (−1.00000, 1.00000, −1.00000)

Vector[38] = (1.00000, 1.00000, −1.00000)

Vector[39] = (1.00000, 1.00000, 0.00000)

Vector[40] = (−0.00000, −1.00000, −1.00000)

Vector[41] = (−1.00000, −0.00000, −1.00000)

Vector[42] = (−1.00000, 1.00000, 0.00000)

Vector[43] = (−0.00000, 1.00000, −1.00000)

Vector[44] = (1.00000, −0.00000, −1.00000)

Vector[45] = (1.00000, −0.11968, −0.22826)

Vector[46] = (0.45391, 1.00000, −0.74490)

Vector[47] = (0.30929, −0.52908, −1.00000)

Vector[48] = (1.00000, 0.32570, −0.82547)

Vector[49] = (−0.53649, −0.28378, −1.00000)

Vector[50] = (1.00000, 0.28277, −0.16662)

Vector[51] = (0.37769, 0.13072, −1.00000)

Vector[52] = (−0.18512, 0.21731, −1.00000)

Vector[53] = (−0.32054, −1.00000, −0.32795)

Vector[54] = (0.37433, 1.00000, −0.18999)

Vector[55] = (−1.00000, 0.49160, −0.79472)

Vector[56] = (−0.09712, 1.00000, −0.03398)

Vector[57] = (−1.00000, −0.00905, −0.41514)

Vector[58] = (0.36282, −1.00000, −0.54229)

Vector[59] = (−0.52123, 1.00000, −0.11772)

Vector[60] = (−0.31196, 1.00000, −0.73018)

Vector[61] = (−1.00000, −0.69636, −0.40808)

Vector[62] = (−1.00000, 0.32257, −0.12697)

Vector[63] = (1.00000, −0.48206, −0.55084)

Vector[64] = (−0.06116, −0.17609, −1.00000)
